# On the Origins of Some Spectroscopic Properties of “Purple Iron” (the Tetraoxoferrate(VI) Ion) and Its Pourbaix Safe-Space

**DOI:** 10.3390/molecules26175266

**Published:** 2021-08-30

**Authors:** Philip C.W. Cheung, Daryl R. Williams, Jack Barrett, James Barker, Donald W. Kirk

**Affiliations:** 1 Department of Chemical Engineering, Imperial College, London SW7 2AZ, UK; d.r.williams@imperial.ac.uk; 2 Department of Chemistry, King’s College, University of London, London WC2R 2LS, UK; jackbarrett273k@aol.com; 3 School of Life Sciences, Pharmacy and Chemistry, Kingston University, Kingston-upon-Thames KT1 2EE, UK; J.Barker@kingston.ac.uk; 4 Department of Chemical Engineering & Applied Chemistry, University of Toronto, Toronto, ON M5S 3E5, Canada; don.kirk@utoronto.ca

**Keywords:** ferrate(VI), ultraviolet-visible, infrared, Raman spectroscopies, Pourbaix diagram

## Abstract

In this work, the authors attempt to interpret the visible, infrared and Raman spectra of ferrate(VI) by means of theoretical physical-inorganic chemistry and historical highlights in this field of interest. In addition, the sacrificial decomposition of ferrate(VI) during water treatment will also be discussed together with a brief mention of how Rayleigh scattering caused by the decomposition of Fe**^VI^**O_4_^2−^ may render absorbance readings erroneous. This work is not a compendium of all the instrumental methods of analysis which have been deployed to identify ferrate(VI) or to study its plethora of reactions, but mention will be made of the relevant techniques (e.g., Mössbauer Spectroscopy amongst others) which support and advance this overall discourse at appropriate junctures, without undue elaboration on the foundational physics of these techniques.

## 1. Introduction

Together with synthetic and analytical chemists, research scientists from the chemical, civil and environmental engineering professions tasked with potable and wastewater treatment have shown keen interest in learning more about the deep dark purple tetraoxoferrate(VI) ion Fe**^VI^**O_4_^2−^ in aqueous solution, hailed as one of the most “ecologically friendly” chemical entities to date. Branded with the sobriquet “Purple Iron”, its advocates are looking forward to demonstrating (scientifically, it is hoped) that it is “greener” than the green chlorine gas and hypohalites in aqueous solution. Research colleagues delved deep into literature covering many subject areas including those of the spectroscopic and thermodynamic properties of Fe(VI) compounds. Their ensuing queries include the causality of the “dark as permanganate” colouration of the Fe(VI) oxidant and the origins of multiple peaks in the Ultraviolet-Visible (“UV-Vis”), Infrared (IR) and Raman spectra related to the Fe**^VI^**O_4_^2−^ ion. In this work the authors endeavour to address these areas of interest.

### 1.1. Examples of the Latest Applications of Ferrate(VI)

For more than two decades, the tetraoxoferrate(VI) ion Fe**^VI^**O_4_^2−^ (aq) has been considered as one of the most environmentally benign oxidizing agents for a multitude of purposes, including the treatment of drinking water and wastewaters. Recently, innovative methods of generation of ferrate(VI) and its applications have been identified. Diaz et al. [1] produced ferrate(VI) in highly alkaline solutions (NaOH or KOH at 10 mol dm^−3^, the theoretical pH at this concentration is 16.15 according to Licht [2]) by electro-oxidation of several inexpensive iron-containing materials (as electrodes) and found that Grey Cast Iron, the alloy composed of Fe-C(graphite)-Si, is the best material. McBeath et al. [3] demonstrated a new concept of the generation of ferrate(VI), in situ, by electrochemical oxidation of Fe^2+^ ions already present in wastewaters at near-neutral pH, the Fe(VI) ions produced in turn oxidizing other co-existing contaminants. An example of recycling-and-reuse of wastes was announced by Munyengabe et al. [4] who illustrated the potential of ferrate(VI)-treated sludge, from acid drainages from coal and gold mines, as a starting material for the synthesis of magnetic nanoparticles. A useful application of ferrate(VI) recently brought to light by Alshahri et al. [5] is the mitigation of shutdowns of water desalination plants, often caused by the fouling of reverse osmosis membranes by algae blooms; the pre-emptive method was the treatment of sea water by ferrate(VI) before filtration. This work is followed by that of Addison et al. [6] who optimized the process of the attack of ferrate(VI) on algae, an informative piece of operational research which focused not only on significant parameters in physical chemistry but also on the influence of fluid dynamics on the number of particle collisions between oxidant and reductant per unit time. The research is a rare combination of surface science and particle mechanics.

Recently, more than a few publications have been added to the genre of proof-of-concepts in the elimination of water contaminants. Interesting examples include the work of Bujannovic et al. [7] who saw the encapsulation of crystalline barium ferrate(VI) in micro-to-nano sized particles in paraffin wax (as a supporting solid matrix) for the destruction of clomazone pesticides in water, and reported that this may be more efficient than redox carried out in the bulk of the solution. Acosta-Rangel et al. [8] eliminated synthetic sulphonamide antibiotics from wastewaters, a necessary step taken to prevent the mutation of bacteria which are resistant to the antibiotics, since these mutants will tend to proliferate in the natural environment if not controlled. Montfort et al. [9] managed to cleanse water and soil of pentachlorophenol by ferrate(VI), which vastly outperformed H_2_O_2_ in this exercise. This juxtaposition between performances of oxidizing agents is noteworthy because it validates the call by Cheung & Williams [10] to the attention of the consideration of chemical feasibility during the choice of treatment or remediation agents. Simply put, there is no guarantee that any waterborne compound will donate an electron into the anti-bonding molecular orbital of the –O-O– bridge in a molecule of hydrogen peroxide HO-OH resulting in its fragmentation (H_2_O_2_ + e^−^ → HO^●^ + OH^−^) under the prevailing physicochemical conditions, otherwise Fenton or Fenton-like reactions have to be resorted to.

The tetraoxoferrate(VI) ion finds itself bathing in limelight (besides aqueous and non-aqueous solvent systems) in the field of sustainable energy lately. Efficient water-splitting at ambient temperatures will be a significant breakthrough in science and technology that could underpin an economy powered by hydrogen. Photosynthesis produces oxygen but not hydrogen, and so the quest is on for catalysts based on transition metal oxides which will mimic the “oxygen-evolving complex” (OEC), which is the cofactor of the “photosystem **II**” enzyme and the site of the photolysis of water during the light reactions of photosynthesis. Research is directed towards the elucidation of mechanisms of the splitting of H_2_O to gain insights needed to manufacture these catalysts. Compared to precious metals such as platinum, iron is inexpensive and the action of Fe(VI) species including Fe**^VI^**O_4_^2−^ on water is currently the subject of intense study [11,12]. Platinum feels the heat of competition [13].

### 1.2. Chemical Feasibility as a Consideration in the Choice of Oxidants

A related issue involving the choice of oxidants needs addressing. When one seeks the aid of ultraviolet radiolysis or Fenton reagents to initiate H_2_O_2_, the extremely short-lived free radical HO^●^ with a half-life of the order of 1 μs is created in situ, which means that “concentrations of the free radicals in aqueous solution will not exceed a concentration of 10^−12^ mol dm^−3^ at any one time [14]”. Ozone-based water treatment systems have managed to produce a concentration of the order 10^−10^ mol dm^−3^, but the concentration also decreases rapidly [15]. Regardless of what the steady-state concentration in a reaction system is, the higher the concentration of a water contaminant (the reductant), the longer it takes for its detoxification by the necessary extent of the redox reaction. The authors of the present work also noticed that research on the behaviour of HO^●^ in highly alkaline conditions is relatively rare. The reaction HO^●^ + OH^−^ → O^●−^ (*aq*) + H_2_O (*l*) becomes increasingly significant at pH > 11, since the pK_a_ of HO^●^ is 11.8 [16]. The species O^●−^ is known as the “atomic oxygen anionic radical” (and has been mistaken for the dioxide anionic radical, the “Reactive Oxygen Species” O_2_^●−^, which is often encountered in university courses of medical biochemistry). To the credit of Dorfman & Adams [17], systematic compilation of a handful of reactions began in 1972, with the comment that the ability of O^●−^ to abstract hydrogen atoms (as the mode of oxidation of some molecules) is only “slightly less” than that of the HO^●^ radical. Lee & Grabowski [18] documented more reactions of O^●−^ and these were published in 1992. It would be useful to know how, and how fast, the radical O^●−^ reacts with a variety of species in dilute solution at pH > 13. This probably demands careful monitoring of the reaction mixture by Electron Paramagnetic Resonance (EPR) spectroscopy. Is it possible to treat an alkaline wastewater just by dissolving solid K_2_FeO_4_ in it? What will be the efficacy of FeO_4_^2−^ (aq) compared to the O^●−^ radical? These questions can only be answered by empirical experimentation and observation. There are even more oxidizing species in the reaction mixture to be considered. Baerends et al. identified the most stable intermediate oxidant in chemical reactions between Fenton’s reagent and a reductant in aqueous solution to be [Fe**^IV^**(H_2_O)_5_O]^2+^ [19]. Soon after, Baerends et al. compared the chemical reactivity of Fenton’s reagent (Fe^2+^/H_2_O_2_) and the Fenton-like reagent ([Fe**^III^**(H_2_O)_5_(H_2_O_2_)]^3^^+^ and they discovered that the former is more active. As an oxidizing agent, the [Fe**^IV^**O]^2^^+^ ion happens to be more reactive than [Fe**^VI^**O_4_]^2^^−^ [20]. This research by Baerends et al. on Fenton’s reaction is a good example whereby kinetic data from reaction systems gain strong support from Density Theory Function (DFT) calculations.

### 1.3. Interesting Case Studies in Instrumental Methods of Analysis for Ferrate(VI)

When a new chemical compound with a high degree of purity is synthesized and a crystal of it is obtained, analysts apply many techniques to examine it in great detail. The data gathered from all these methods usually merge to become a collective tool for identification, further inquiries and more analysis. For example, when Planet Earth entered into a new millennium in 2000, Tsapin et al. (NASA) proposed ferrate(VI) to be an oxidizing agent arthropogenic to Planet Mars and responsible for any redox reaction which took place in soil on the surface [21]. In addition to all the analytical work on compounds of ferrate(VI) reported in literature at the time, more spectral data were acquired. These included UV-Vis absorption spectra, visible and infrared reflectance spectra, X-band Electron Paramagnetic Resonance (EPR) spectra, Mössbauer spectra and X-Ray absorption near-edge spectra (XANES). To complete the list, hysteresis loops were measured in magnetic fields up to 1.5 Tesla by a vibrating magnetometer. (Polemics soon followed, not about the choice of instrumental methods of analysis, but of the hypothesis of the existence of Fe(VI) species on Mars [22,23]). Another instance which involved the analysis of ferrate(VI) by a large array of analytical methods instigated by Licht et al. is noteworthy [24]. When the storage of energy was made possible by cathodic Fe(VI) compounds in electrochemical cells, their performances were monitored by a number of spectral techniques such as UV-Vis, FTIR (Fourier Transform Infrared), ICP (Inductively Coupled Plasma) and XRD (X-ray Powder Diffraction). Galvanostatic, potentiometric and cyclic voltammetric methods were also deployed, together with a titrimetric technique which depends on the oxidation of Cr(III) to Cr(VI), by Fe(VI). Physical methods included the use of electrochemical discharge probes. All contributed to the understanding of the long-term performance of these Super-Iron Batteries [24].

## 2. Literature Survey

### 2.1. Instrumental Methods of Analyses Available to Research Activities

Chemical, Civil and Environmental Engineering graduates who enter into academic and/or industrial research for the first time are often required to learn the fundamental concepts and instrumental methods of chemical analysis quickly, and this has been a challenge to many [25]. The Canadian Journal of Chemical Engineering has been publishing, since 2018, a series of review articles (technical notes) each introducing a specific instrumental method of analysis utilized in research. The writing style is informative and the series is updated continually [26]. As an illustration of its overarching pedagogic strategy, the contributing group of Rocha et al. [27] performed a bibliometric analysis of the 10,000 most cited publications between 2016 and 2017 for research teams which had deployed “UV-Vis” spectrophotometry. Collectively, the data shows that research activities involving this method fell within four main categories:

1. Nanoparticles and nanostructures.
2. Biological interactions of silver and gold nanoparticles.
3. Water treatment and Photocatalysis.
4. Examination of crystals, complexes and derivatives.

With Fourier Transform Infrared (FTIR) Spectroscopy [28], two related fields of interest appeared, namely, “aqueous solutions” and “wastewater”. With Raman Spectroscopy [29], the umbrella term “physical chemistry” appeared in the keywords. Other instrumental methods of chemical analysis contained in the series of reports mentioned above include Electron Paramagnetic Resonance (EPR/ESR) Spectroscopy [30], Nuclear Magnetic Resonance (NMR), Mass Spectrometry (MS), X-Ray Photoelectron Spectroscopy (XPS), amongst others. In the series, there is also a paper introducing fluorescence emission spectroscopy [31].

Mössbauer Spectroscopy can provide precise information about the chemical, structural, magnetic and time-dependent properties of a material. The “Mössbauer effect” (of which an alternative name is “nuclear gamma resonance fluorescence”) is a nuclear phenomenon discovered by the eminent German physicist Rudolf Ludwig Mössbauer in 1957 at the University of Munich when he investigated the resonance scattering of gamma photons (129 keV) emitted by the excited ^191^Ir nuclei, formed during the beta decay of the mother isotope ^191^Os. The Mössbauer effect has been observed with 42 elements; its main use, however, is in the study of iron and tin compounds. Both Mössbauer and ESR spectroscopies have been utilized in ferrate(VI) research and the results have been reviewed recently. In their review paper, Luo et al. included Mössbauer Spectroscopy in the toolkit for ferrate(VI) research [32], citing as an example the monitoring of the aging of freshly prepared crystalline ferrate(VI) via the appearance of isomer shifts of lower oxidation states which may have made a brief appearance according to the reduction sequence Fe(VI) → Fe(V) → Fe(IV) → Fe(III). Not too many complexes of iron in the +4 oxidation state have been reported in the literature since their stabilities are highly sensitive to surrounding conditions, although Sr_2_FeO_4_ and Ba_2_FeO_4_ have long been known [33]. These ferrates do not contain discrete ions of [Fe**^IV^**O_4_]^4−^, but are mixed oxides, with barium ferrate(IV) crystals having a spinel structure [33]. Is it possible that these ferrates are non-stoichiometric? A gap of knowledge exists and requires investigation. Nevertheless, Karolína Machalová-Šišková et al. reported the successful synthesis of the two crystals Na_4_Fe**^IV^**O_4_ and K_3_Fe**^V^**O_4_, confirmed by Mössbauer spectroscopy [34]. The stability of crystalline K_2_FeO_4_ in air was monitored by Machala et al. with the same technique [35] and the trend of Mössbauer analysis continued unabated. With Mössbauer spectroscopy, Sharma et al. [36] studied the kinetics of the reaction between ferrate(VI) with sulfamethoxazole and aniline in alkaline solutions. Sharma et al. [37] also reviewed the Mössbauer work which addressed the stability of the +4, +5 and +6 oxidation states of iron from the point of view of monitoring ferrate(VI) decomposition, with many examples of real conditions under which solid ferrates are prone to decomposition. To physical-inorganic chemists, whose expertise are in aqueous systems, this rare glimpse of solid-state reactions between Fe_2_O_3_ and Na_2_O to produce ferrates is relevant to their overall understanding of the subject matter. Mössbauer spectroscopy is indispensable in the study of iron compounds. The publications mentioned in this paragraph ([32,33,34,35,36,37]) which include two detailed reviews will be most informative to those researchers using the technique. The literature on the subject, of course, is prolific.

However, as far as the paramagnetic ferrate(VI) is concerned, NMR may not be the best method for the identification and confirmation of the presence of the Fe(VI) ion. The natural sensitivity of iron towards NMR is extremely low and therefore NMR is not a good candidate for the direct observation of ^57^Fe. The spectral range (measured in NMR in the unit of “ppm”) is also extremely wide (9000 ppm) and to complicate the issue, paramagnetic species lose their magnetization very rapidly (via T2 relaxation pathways). These phenomena result in further losses in sensitivity (i.e., losses in peak intensity) and also in resolution (i.e., losses in peak width). This means that observing paramagnetic iron species is impossible, especially if they are not bound to a ligand that might have otherwise allowed indirect observation of the iron centre, by using a correlational spectroscopy.

The situation with iron is certainly not the same as the tetraoxo anions of some other transition elements. Ziegler et al. [38] analysed the NMR shielding constants, obtained experimentally, of the following ions: Cr^(+**VI)**^O_4_^2−^ and Mn^(+**VII)**^O_4_^−^, their 4*f* congeners: [Mo^(+**VI**)^O_4_]^2−^, [Tc^(+**VII**)^O_4_]^−^, Ru^(+**VIII**)^O_4_ and their 5*f* homologues: [W^(+**VI**)^O_4_]^2−^, [Re^(+**VII**)^O_4_]^−^, Os^(+**VIII**)^O_4_. The application of “Double Perturbation Theory” has proven to be a powerful method in the toolkit of computational chemistry. Direct measurement of magnetic moment of Fe(VI) is also intrinsically difficult. Simply, the Fe atom in ferrate(VI) has a 3*d*^2^ electronic configuration, with the two unpaired electrons occupying the two degenerate and low-energy states generated by four O_2_^2−^ ions in a tetrahedral crystal field, resulting in paramagnetism. The depiction of the magnetic moment of ferrate(VI) by Schmidbaur (2018) by means of Crystal Field Theory (CFT) is clear and succinct, “For this ^3^*A*_2_ ground state no significant orbital contributions are expected and a spin-only value for the magnetic moment should be valid. A spin-orbit coupling would lower the magnetic moment if such coupling with the upper ^3^*T*_2_ states is considered, which is separated by the crystal field splitting energy 4.45 *Dq* estimated at −12.720 cm^–1^” [39].

As far as analytical methods based on magnetism are concerned, the best to investigate Fe(VI) is EPR (electron paramagnetic resonance spectroscopy, formerly called electron spin resonance, ESR). Carrington et al. obtained the spectra from single crystals of potassium ferrate (VI) which were free from impurities [40,41].

By applying the Hamiltonian energy operator for spin states, an isotropic (i.e., having the same value in all Cartesian directions *x*, *y* and *z*) “*g* factor” of *g* = 2, at spin quantum number S = 1, was arrived at (with *g_x_ = g_y_ = g_z_*). EPR investigates electrons with both orbital and spin angular momentum, and therefore requires the introduction of a scaling factor to address the coupling between the two. This factor is called the “*g*-factor” (and it resembles the way in which the “chemical shift” is used in NMR). With unpaired electrons, inferences can be made from free radicals. In the literature, one encounters the value of the *g*-factor as “2.0023” on numerous occasions. It so happens that the *g*-factor for most free radicals is very close to this numerical value, since the unpaired electron has precious little orbital contribution to the magnetic moment (e.g., spin-orbit coupling is minute in most carbon-based radicals). The result of *g* = 2 obtained by Carrington et al. can only mean that the ground state ^3^*A*_2_ and the first excited state are separated so that spin-orbit coupling is rendered insignificant [40,41]. The findings of Carrington et al. [40,41] in 1956–1960 were certainly not lost on Schmidbaur [39] in 2018. Schmidbaur (2018) also reported anti-ferromagnetism being exhibited by potassium, strontium and barium ferrates(VI) when cooled to the temperature of liquid helium, a phenomenon observed by Mössbauer spectroscopists [39].

**Figure 1:**
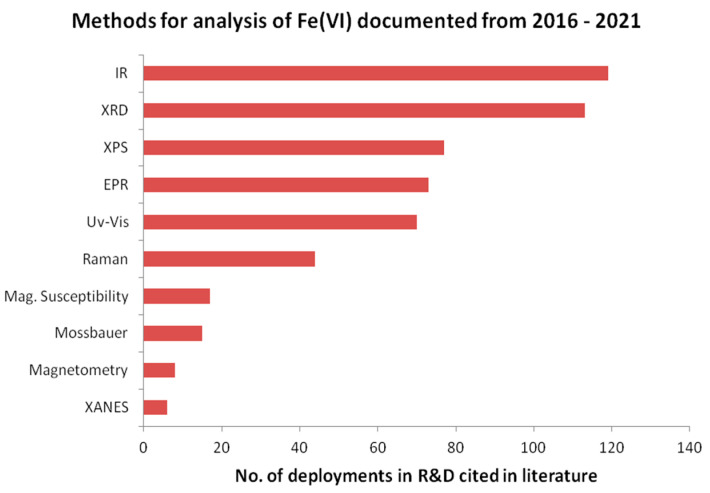
Methods of analysis of Fe(VI) moieties documented in literature sampled between 2016–2021. (Spectral methods: IR = infrared/reflectance/Fourier Transform, XRD = X-ray diffraction, XPS = X-ray photoelectron spectroscopy, EPR = electron paramagnetic resonance, UV-Vis = ultraviolet & visible, followed by Raman spectroscopy, Magnetic Susceptibility, Mössbauer spectroscopy and XANES = X-ray absorption near-edge spectra).

Spectroscopic methods have been indispensable tools for the identification of ferrates, their decompositions and their redox reactions. The whole corpus of scientific publications reported on spectroscopic studies of metal and ammonium ferrates(VI) as crystals and in aqueous solutions is immense and expanding (timeline: the X-ray crystal structure of K_2_FeO_4_ has been known for 70 years and electronic crystallographic libraries are huge). Literature data from the search engine ScienceDirect (www.sciencedirect.com, accessed on 30 June 2021) are extracted. The data consists of the types of instrumental methods of analysis and the number of times they appeared in publications, and plotted in Figure 1. To keep the volume of information manageable, the survey was carried out for the literature published in the period 2016–2021, and this sample shall suffice for the sake of illustration of the popularity of these spectral methods. (There are other search engines for literature but the data will no doubt overlap). The authors also envisage publishing another detailed review paper on Mössbauer spectroscopy based on the relevant fundamental concepts and equations of the nuclear physics on which the spectroscopy is based.

### 2.2. The Tetraoxoferrate (VI) Ion

The present review focuses on UV-Vis, IR and Raman spectroscopies. With regards to the origins of these spectra, the authors wish to describe the visible spectrum of ferrate(VI) in terms of the Molecular Orbital (MO) Theory of the iron-oxygen bond, and the infrared (IR) and Raman spectra of solid and aqueous ferrate(VI) in terms of aspects of Group Theory. In addition to spectral properties, the thermodynamic stability of ferrate(VI) in aqueous solutions as a function of pH is also discussed using the Pourbaix (pH-E_h_) diagram for iron.

The conceptualization of the electronic structure and bonding of the [Fe**^VI^**O_4_]^2−^ moiety as a resonance hybrid (of three canonical structures) took time to mature. X-ray powder diffraction patterns were first obtained in the 1950s and revealed that the three-dimensional geometry of the ferrate ion in K_2_FeO_4_ crystals is tetrahedral [42,43].

**Figure 2:**
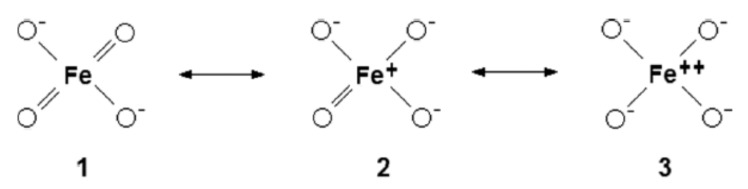
The resonance hybrids of the doubly negative-charged tetraoxoferrate(VI) ion [47].

In 1966, Griffith obtained the infrared spectrum of ferrate in alkaline solution and concluded that the anion remained tetrahedral in aqueous environment [44]. In 1971, Goff & Murmann demonstrated, by an ^18^O exchange technique, that all four oxygen atoms are identical, kinetically speaking [45]. From early efforts to elucidate the mechanisms of oxidation of alkenes by chromyl chloride [46], permanganate and osmium tetroxide, it became known that these inorganic metal-oxygen species are so heavily polarized that the molecular moiety with multipoles can be represented schematically as: L_n_M=O ⇌ L_n_M^+^−O^−^ (i.e., two structural forms in equilibrium; in the former structure, the oxygen atom is double bonded to the metal). In 1997, these developments led Norcross et al. [47] to posit that, “the ferrate ion is best visualized as a positively charged iron cation surrounded by four equivalent oxygens, each bearing a partial negative charge; i.e., a resonance hybrid of three canonical structures, **1**–**3**”, as shown in Figure 2.

Norcross et al. [47] then asserted that structure **3** (in Figure 2) is probably the most prominent, based on a theoretical study of tetrahedral, tetraoxo anions of the transition elements carried out by Ziegler et al. [48]. Employing the Hartree-Fock-Slater Discrete Variational Method, they predicted that the profile of the d-orbital populace, regardless of d^0^, d^1^ or d^2^, will be very similar to those of the corresponding divalent cation M^2+^ in which two electrons have been ejected from the (*n* + 1)s orbital of M, the metallic element. Although Ziegler et al. [48] did not involve [FeO_4_]^2−^ specifically in their analysis (the tetra-oxo moieties examined were [MnO_4_]^−^, [MnO_4_]^2−^, [MnO_4_]^3−^, [CrO_4_]^2−^, [CrO_4_]^3−^, [VO_4_]^3−^, RuO_4_, [RuO_4_]^−^, [RuO_4_]^2−^, [TcO_4_]^−^ and [MoO_4_]^2−^), their argument is so convincing that the generalization made it possible for Norcross et al. [47] to favour structure **3** as the most significant, as far as redox reactions are concerned. In 1994, Jitsuhiro et al. chose the Symmetry Adapted Cluster (SAC) and the SAC-configuration interaction (SAC-CI) theories as tools for their analysis of the [CrO_4_]^2−^ ion in the ground and excited states [49]. The analysis resulted in the positive charge of the central metal ion taking on values between 1.04 and 1.44, prompting Norcross et al. [47] to favour both structures **2** and **3** as candidates over structure **1** in Figure 2. The Hartree-Fock-Slater Discrete Variational Method, SAC and SAC-CI theories mentioned immediately above could have been applied to analyse [FeO_4_]^2−^ in its ground and excited states with an aim of gaining the same insights into structure and bonding. After all, laboratory preparations of metal ferrate(VI) both as crystals and in aqueous solutions, together with their spectra, have been reported in the literature since the 1950s. The canonical structures in Figure 2 (adapted from [47]) never went out of fashion amongst authors. For example, Ghernaout & Naceur [50] used the same diagram to illustrate the nature of bonding in [Fe^VI^O_4_]^2−^ while reviewing its many uses in 2011; in 2016, Talaiekhozani et al. discussed the structure and bonding of ferrate(VI) before reviewing all the methods for the production of ferrate(VI) [51].

## 3. Molecular Orbital Theory Applied to Ferrate(VI)

### Symmetry and Bonding in Ferrate(VI)

Freshly prepared aqueous solutions containing 0.1 mol dm^−3^ of FeO_4_^2−^ ions at pH = 14 will remain stable in refrigeration (0 °C < T °C ≤ 3 °C) for prolonged periods of time up to a fortnight (and may be stable for perhaps longer), before decomposing to the orange/red Fe(III) “hydroxides”. These alkaline solutions are heavily laden with solutes and therefore do not freeze at 0 °C due to their cryoscopic property. The colouration of these alkaline solutions is akin to that of an opaque, deep purple solution of KMnO_4_. In aqueous solution, ions achieve thermodynamic stability to a large extent by their hydration enthalpy. This exothermic term must out-balance any expenditure of energy in oxidising the metal to its particular oxidation state. This is usually the case for ionic charges up to +3. These lower oxidation states are normally octahedrally coordinated by water molecules. For higher oxidation states, stability can be achieved only by the formation of metal-oxygen π-bonds. Such bonding reduces the formal charge on the metallic element and (usually) produces negatively charged ions which benefit from hydration enthalpy [52].

An unprotonated and undistorted Fe**^VI^**O_4_^2−^ ion with *d*^2^ tetrahedral geometry can be assigned the point group *T_d_* and belongs to a very small group of similar transition metal ions, one of which is Ni^0^(CO)_4_ with tetrahedral geometry and also belongs to the *T_d_* point group. There has been consensus that iron has reached a maximum oxidation state of +6, but in 1994 Schröder et al. bombarded a mixture of O_2_ and Fe(CO)_5_ (in the mole ratio of 100:1) with 100 eV electrons and obtained the species [FeO_4_]^−^ as discrete entities in the gaseous phase [53]. In 2016, Lu et al. blasted Fe atoms off of a piece of rotating iron with a laser beam and then deposited them, together with O_2_ molecules and electrons, onto a solid caesium iodide matrix maintained at a temperature of 4 Kelvin [54]. Stoichiometrically, the reaction is: Fe + 2O_2_ + e^−^ → [FeO_4_]^−^. The isotopes ^16^O and ^18^O were used in the synthesis to allow infrared monitoring of isotopic kinetic effects. The product [FeO_4_]^−^ was then subjected to UV-Vis radiation and the resulting reversal of two asymmetric vibrational frequencies *ν*_asy_(Fe-O) suggested the possibility of two isomers. From Density Function Theory (DFT) and Wave Function Theory (WFT) calculations, four low-energy and near-degenerate structures were hypothesized with one of them being a true tetroxide. The evidence for the existence of a stable molecular ion constituted of iron in its +7 oxidation state has not been refuted.

There are nine *d*^0^ tetrahedral tetraoxo transition metal complexes (with empty *e* orbitals) which have been studied extensively, their spectral and physical-chemical properties well documented. The oxidation states of the central metal ion in each of the nine complexes have reached their highest oxidation state known to the element. They are:

1st row elements: V^(+**V)**^O_4_^3−^, Cr^(+**VI)**^O_4_^2−^ and Mn^(+**VII)**^O_4_^−^. Note that, in aqueous solutions, Mn**^V^**O_4_^3−^ and Mn**^VI^**O_4_^2−^ are extremely unstable at any pH. Both disproportionate into Mn**^VII^**O_4_^−^, and to Mn^2+^ (in acidic solution), or to Mn**^IV^**O_2_ (in alkaline solution).
2nd row elements: [Mo^(+**VI**)^O_4_]^2−^, [Tc^(+**VII**)^O_4_]^−^, Ru^(+**VIII**)^O_4_.
3rd row elements: [W^(+**VI**)^O_4_]^2−^, [Re^(+**VII**)^O_4_]^−^, Os^(+**VIII**)^O_4_.

In 2012, Ziegler et al. investigated the electronic absorption spectra of MnO_4_^−^, TcO_4_^−^, RuO_4_ and OsO_4_ using time-dependent Density Function Theory (TDDFT) and Frank-Condon Theory (FCT) [55]. This exercise is the first study of its kind on the symmetry of excited states of tetraoxo *d*^0^ transition metal complexes by computation. Ziegler et al. [55] stated that “The many computational studies reveal that a theoretical description of the tetraoxo complexes is more difficult and challenging than one might expect based on their modest size and high symmetry. Nevertheless, consensus has been reached as to the assignment of the electronic spectra for the tetraoxo complexes with the possible exception of permanganate. The latter complex is exceptionally difficult to describe, even in the ground state”. The exercise is a comparison of the outcomes of the application of a calculation scheme developed by Tom Ziegler et al. [56] to the four named tetraoxo ions with a focus on the resolution of computational issues surrounding permanganate. The results are useful. In the ground state, all the tetraoxo ions show *T_d_* symmetry. In the excited states, all complexes demonstrate Jahn-Teller distortions away from *T_d_* and adopt lower symmetries. (See also [57]). The first excited state for all complexes has *C*_3*V*_ symmetry and is owed to a charge transfer between the oxygen HOMO (Highest Occupied Molecular Orbital) to the metal LUMO (Lowest Unoccupied Molecular Orbital), a 1t_1_ → 2e transition. The second excited states of TcO_4_^−^, RuO_4_ and OsO_4_ exhibit *D*_2*d*_ symmetry and is owed to charge transfer from the oxygen HOMO-1 to the metal LUMO, with MnO_4_^−^ taking on two *C*_2_*_v_* geometries. The excitation for all four complexes is: 2t_2_ → 2e. The third excited state in MnO_4_^−^ resulted from a HOMO to metal LUMO+1 charge transfer, a 1t_1_ → 3t_2_ transition which resulted in *D*_2*d*_ symmetry. Based on these calculations, the Frank-Condon method was used to mimic the vibronic structure of the absorption spectra of the complexes. The Frank-Condon calculations “seems to reproduce the salient features of experimental spectra as well as the simulated vibronic structure for MnO_4_^−^ generated from an alternative scheme that does not apply the Franck−Condon approximation” [58].

Vibronic spectroscopy is a type of molecular spectroscopy concerned with the simultaneous changes in electronic and vibrational energy levels of a molecular entity due to the absorption or emission of a photon with the right amount of energy. The intensity of vibronic transitions is governed by the Franck–Condon principle. One other piece of important information that vibronic spectroscopy can provide is bond length, changes in which have implications for mechanism elucidation for chemical reactions.

The late Professor Tom Ziegler (1945–2015) at the University of Calgary (Canada) published his last paper on the applications of time-dependent and time-independent Density Theory Function (DFT) calculations on all the nine *d*^0^ tetraoxo transition metal complexes, from all three transition series [59], listed above. We wait to see how the electronic transitions of ferrate(VI) moieties can be explained by computational chemistry.

The Fe**^VI^**O_4_^2−^ ion has two unpaired *d* electrons (it is a high-spin, paramagnetic complex) with an *sd*^3^ hybridized iron centre. Other tetraoxo ions with a *d*^2^ configuration are Mn**^V^**O_4_^3−^ (*T_d_* with a triple ground state [60], and is bright blue), Cr**^VI^**O_4_^2−^ and Ru**^VI^**O_4_^2−^. It is noteworthy that not all Fe(VI) compounds have tetrahedral symmetry. Berry et al. [61] synthesized the Fe(VI)-nitrido complex with formula [(Me_3_cy-ac)Fe**^VI^**N](PF_6_)_2_ and its octahedral symmetry was confirmed by Mössbauer, X-ray absorption (EXAFS) and IR spectroscopies, together with Density Function Theory (DFT) calculations. With the nitrido complex, the Fe**^VI^** ion is positively charged. Unlike the Fe**^VI^**O_4_^2−^ ion which is paramagnetic, the nitrido compound is diamagnetic. Generally, as a consequence of the 1st Laporte selection rule which forbids *d-d* transitions in a complex with a centre of symmetry, absorption bands in octahedral complexes are weak (low molar absorptivities). On the contrary, the absorption bands of tetrahedral complexes are more intense. Note that before the term “molar absorptivity” was adopted, the term “molar extinction coefficient” was used in earlier literature.

In aqueous solutions, the vast majority of transition metal ions possess octahedrally coordinated water molecules of hydration, e.g., [Fe**^II^**(H_2_O)_6_]^2+^. A relatively small fraction of transition metal ions possesses square-planar symmetry, and an even smaller fraction has tetrahedral symmetry, of which Fe**^VI^**O_4_^2−^ is one. Tetrahedral symmetry seems to occur as the result of excessive inter-ligand repulsion. For example, the Fe**^III^**Cl_4_^−^ ion is tetrahedral because the relatively large and negatively charged chloride ligands minimize their mutual repulsion by adopting tetrahedral geometry rather than the electronically preferred square-planar symmetry. In the cases of tetrahedral oxo-ions, the mutual repulsion between the oxygen ligand atoms seems to be the most dominating factor in stabilizing their symmetry. In crystals, the presence of metal cations will distort the tetrahedral symmetry to various degrees.

**Figure 3:**
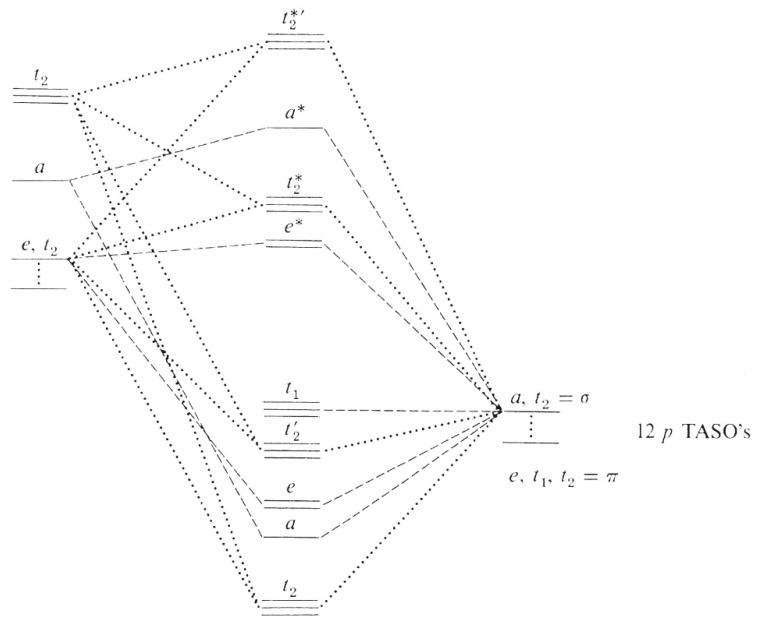
Molecular Orbital scheme: MD_4_ (*T_d_*) orbital energies showing both *σ* and *π* interactions [63]. (TASO stands for Terminal Atom Symmetry Orbital).

The bare “Fe^6+^ ion” has electronic configuration [Ar]3*d*^2^ (and is in the 3*d*^2^, ^3^*F* state). The Fe**^VI^**O_4_^2−^ ion has a *d*^2^ configuration and according to the energy-level order of the Molecular Orbital Theory [62], both *d* electrons occupy the degenerate anti-bonding 2e* orbitals in the ground state, as shown in Figure 3. Each of the two levels of the e* orbitals, shown in Figure 3, is populated by an unpaired *d* electron. These anti-bonding molecular orbitals (ABMOs) of *e* symmetry are derived from the *d*(z^2^) and the *d*(x^2^–y^2^) atomic orbitals (AOs) of the iron atom. Above the e* set in energy scale, there exists an empty set of triply degenerate anti-bonding MOs of *t*^2^ symmetry derived from the *d*(xy), *d*(xz), *d*(yz) atomic orbitals (AOs) of the iron atom. Indeed, the *e* and *t*_2_ orbitals share a predominantly *d* character [63]. By electron spin resonance (ESR) spectroscopy, Carrington et al. ([41,64]) have confirmed that the ground state of Fe**^VI^**O_4_^2−^ is ^3^A_2_ with configuration *e*^2^*t*_2_^0^.

There are 26 valence electrons in the Fe**^VI^**O_4_^2−^ ion (eight from the iron atom, four 2*p* electrons from each of the four oxygen atoms plus two more to make the overall ionic charge to be −2), so the electronic configuration is: (1t_2_)^6^(1a)^2^ (essentially contributing the eight electrons for the four sigma-type bonds between the iron atom and the four oxygen atoms), (1e)^4^(2t_2_)^6^ (contributing to the relatively weak π-bonding, or “partial double bonds”, a term used in the literature occasionally), (1t_1_)^6^ (six non-bonding electrons on the oxygen atoms), (2e)^2^ (the two unpaired anti-bonding electrons). These results are consistent with the tetrahedral geometry of the Fe**^VI^**O_4_^2−^ ion in which the bonding corresponds to *d^3^s* hybridization of the atomic orbitals of the anion. Any further addition of electrons would occur in the 2e and 3t_2_ anti-bonding levels and would contribute to the general instability of the ion, if indeed the reduction of Fe(VI) happens this way.

Generally speaking, for transition metal complexes, energies of the orbitals of ligands involved in ligand-to-metal π-bonding are lower than those of the metal’s t_2g_ orbitals. Therefore, when π interactions occur, it is the ligand’s orbitals which contribute to the lower (bonding) orbitals predominantly. The higher (anti-bonding) molecular orbitals (MOs) are of energies much higher than those of the original t_2g_ orbitals, and should really be labelled as t_2g_*. As a consequence, the e_g_*–t_2g_* energy gap is reduced.

Towards the end of the last millennium, the group of Professor Zoila Barandiaran (Universidad Auto’noma de Madrid, Spain) shed light on the complexity of the interactions between the atomic and molecular orbitals of the five atoms of the Fe**^VI^**O_4_^2−^ ion [65]. Their theoretical calculation adopts an approach called “multi-configurational self-consistent field (MCSCF)”. It is a method for the generation of qualitatively precise reference states of molecules, specifically for moieties where the Hartree-Fork Self-consistent Field Theory and the Density Function Theory, on their own, are not adequate for their descriptions (e.g., in situations where ground states are quasi-degenerate with low-lying excited states, or in scenarios involving the fracturing of chemical bonds). The MCSCF method uses a linear combination of Configuration State Functions (CSF), or configuration determinants, in order to make approximations of the exact electronic wave function of an atom or molecule. The set of MCSCF methods have been known to model highly complex species, reliably calculating their ground and excited states. As the number of CSFs rapidly increases with the number of active orbitals along with computational cost, it may be necessary to use smaller sets of CSFs by restricting the number of electrons in certain subspaces, performed in the Restricted Active Space SCF (RASSCF) method. Here, one allows only single and double excitations from some strongly occupied subset of active orbitals, or restricts the number of electrons in another subset of active orbitals to a maximum of two. However, the RASSCF exercise with the isolated Fe**^VI^**O_4_^2−^ ion ignores any further constraints on the symmetry and energy levels due to the presence of solvents or lattices (i.e., of any electromagnetic force field and their spatial propagation). In fact, they offered the opinion that “the ferrate ion is a good example where Ligand Field Theory and ab initio quantum chemical calculations give completely different interpretations of its bonding and spectroscopic properties”. The next challenge is to explain the experimentally obtained IR/Raman spectra of a real alkaline solution of sodium ferrate(VI) by extending the *ab initio* calculations performed over a single FeO_4_^2−^ ion isolated in space to the same ion in aqueous solution.

## 4. Electronic Spectra

### 4.1. The Absorption Spectrum of Ferrate(VI)

**Figure 4:**
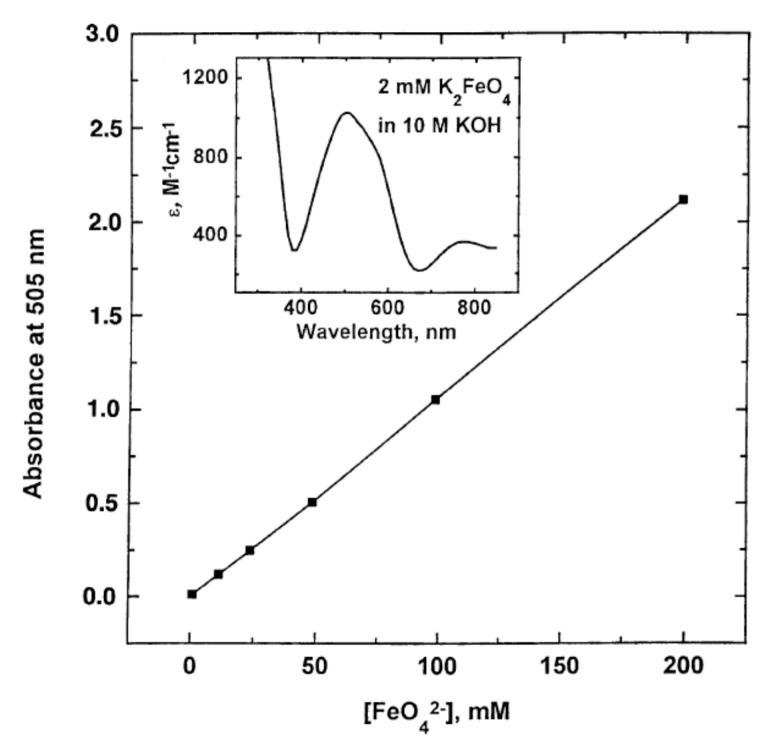
A visible spectrum of K_2_FeO_4_ in alkaline solution showing the 505 nm peak [24].

It is generally the case that many electronic transitions are expected in the complexes of metals with high oxidation states and ferrate(VI) is no exception. The higher the oxidation state, the higher the crystal field splitting energy and many transitions are possible. The visible spectrum of a 2 mM K_2_FeO_4_ solution in 10 M KOH was obtained by Licht et al. [24], Figure 4. (The spectrum is typical of similar preparations and measurements in the literature). In the same graph, the linear Beer-Lambert relationship between solute concentration and absorbance in dilute solutions is also demonstrated. Licht et al. calculated the absorption coefficient to be ε = 1070 ± 30 M^−1^ cm^−1^ at 505 nm [24]. Ligand-to-metal charge transfers quench the highly charged metallic ion and tend to stabilize the entire complex. The deep dark purple colouration of Mn**^VII^**O_4_**^−^** due to the absorption at 528 nm is a ligand-to-metal charge transfer band; an electron from oxygen’s lone pair has been transferred to a low-lying Mn orbital. The spectra in solution must also show charge-transfer-to-solvent bands which have large absorptivity values and are usually observed in the UV regions.

Various workers have used different absorption peaks in the visible region as a means to identify the presence of ferrate(VI), and this approach has led to some confusion in literature. The work of Carrington et al. [64], Guenzburger et al. [66] and Lever [67] concluded that the ion shows extensive charge transfer behaviour, absorbing near 12,700 cm^−1^ (787 nm), which can be ascribed to a *d-d* transition. The *d-d* transition has been assigned to the ^3^A_2_ → ^3^T_1_ change in electronic states, although later authors (Di Sipio et al., [62]) suggested an alternative assignment. In 9 M potassium hydroxide solution, the ferrate(VI) ion exhibits bands at 12,600 cm^−1^ (794 nm, with molar absorptivity ε = 400 M^−1^ cm^−1^), 17,200 cm^−1^ (582 nm, no ε value quoted), 19,800 cm^−1^ (505 nm, ε = 1070–1150 M^−1^ cm^−1^ have been reported depending on pH. These are high absorption coefficients for *d-d* transitions and must mean that the signature band at 505 nm is a ligand-to-metal charge transfer band. A similar conclusion applies to the band at 29,400 cm^−1^ (340 nm, ε = 1100 M^−1^ cm^−1^). Wood [68] reported seeing a broad maximum at around 500 nm (ε = 1070 M^−1^ cm^−1^), together with two minima, one at 390 nm (ε = 318 M^−1^ cm^−1^), the other at 675 nm (ε = 211 M^−1^ cm^−1^). Wood [68] also mentioned that the values of absorption coefficients reported by Kaufman & Schreyer [69] were higher due to the presence of “15% Fe(OH)_3_ in their solutions”.

For a thorough treatment of the structure and reactivity of the oxoanions of transition metals, see Carrington & Symons ([70,71]).

### 4.2. The Orgel Diagram for Tetrahedral Complexes

**Figure 5:**
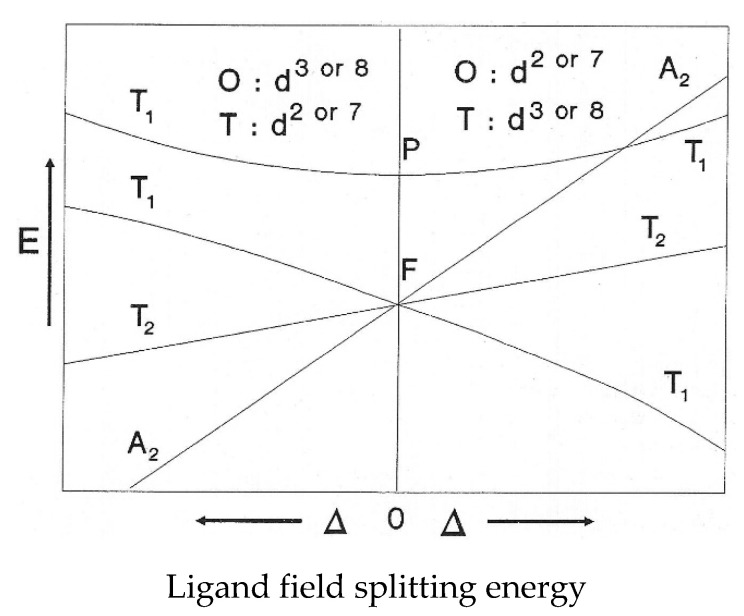
Orgel diagram of *T_d_* and *O_h_* complexes with two, three, seven (high spin) or eight *d* electrons [72].

The Orgel diagram in Figure 5 [72] is divided into two portions by a vertical line in the middle. The compartment in the left-hand side is reserved for complexes with tetrahedral coordination geometry of *d*^2^ configuration, such as Fe**^VI^**O_4_^2−^. The diagram indicates that there should be three *d-d* transitions in a *d*^2^ tetrahedral ion in the order of their increasing energies, namely: ^3^A_2_ → ^3^T_2_, ^3^A_2_ → ^3^T_1_(F), and ^3^A_2_ → ^3^T_1_(P), representing the absorption at 794 nm, the two absorptions at 582 and 505 nm (suggested by Lever [67] to be associated with the same *d-d* transition), and the absorption at 340 nm. The absorption coefficients are all fairly large for *d-d* transitions in a tetrahedral ion and are presumed to be deriving some intensity from the ligand-to-metal charge transfer transition in the ultraviolet region. The absorption in the centre of the visible region, from 582 to 500 nm (*c*.*f*. the broad maximum at 500 nm observed by Wood [68]) is responsible for the purple colour of the ion in solution, red and blue light being transmitted, and the 510 nm peak reported by Sharma [73] fell within this range (in addition to a perceivable shoulder peak between 275 and 320 nm).

Note that in Figure 5, **P** and **F** on the figure refer to the atomic electronic states in the presence of a spherical ligand field. The two d electrons have ***l*** (the secondary quantum number, little ***l***) values of 2 because they are d electrons. The possible combinations of values of l and m with the two electron spins parallel give rise to electronic states with (capital) **L** = 2 or 4, **P** or **F**. The **F** state is the ground state while **P** is the first excited state. There are other states which are singlets (spin-paired) that are not relevant to the spectra since only those transitions with no change in multiplicity are allowed. The symbol delta (Δ) refers to the magnitude of the ligand field splitting energy, the one to the left refers to what happens in a tetrahedral field to a *d*^2^ ion, e.g., in the case of the Fe**^VI^**O_4_^2−^ ion; the right side being the change in splitting that occurs if the *d*^2^ ion is in an octahedral field.

In an aqueous redox system where Fe**^VI^**O_4_^2−^ ions are being depleted during the course of the reactions, their detection by way of the 505–510 nm peak is not always satisfactory. At very low concentrations, the residual anions are difficult to detect because the molar absorptivity ε at that particular wavelength is relatively low to begin with, ε = 1.1 × 10^3^ M^−1^ cm^−1^ (see comparison below). There are two remedies for this and both happen to be spectrophometric methods ([74,75]). The first is to react a colourless solution of the compound 2,2-azino-bis(3-ethylbenzothiazoline-6-sulfonate), better known as ABTS, with a sample of the reaction solution. The green radical cation ABTS^●+^ is formed which absorbs at 415 nm, with ε = 3.40 × 10^4^ M^−1^cm^−1^, an order of magnitude higher than that of the 505/510 nm peak. The method was established at pH = 4.3, and the lowest concentration detected was 0.03 μM. The second method is to mix potassium iodide solution with a sample of the reaction solution. Iodide ions are oxidized to yellowish I_3_^−^ ions which absorbs UV photons at 351 nm (ε = 2.97 × 10^4^ M^−1^cm^−1^). The lowest concentration detected was 0.25 μM, and the working pH range was 5.5 to 9.3, a manageable range for pH adjustment manoeuvres in the laboratory.

## 5. Vibrational Spectra

In solid K_2_FeO_4_ the geometry of the ferrate(VI) ion has been established as tetrahedral from X-ray powder diffraction studies, from 1950 onwards ([42,43]). It became difficult for crystallographers to refrain from assigning the anion to the *T_d_* point group, which is that of the tetrahedral methane CH_4_. The implicit assumption that the Fe**^VI^**O_4_^2−^ ion belongs to the *T_d_* group is prevalent in the early days of study of tetraoxo ions. It has to be emphasized that the point group symbol *T_d_* relates to symmetry, but not a shape. The molecules of CHCl_3_ and CCl_4_ are both tetrahedral in shape, but the former belongs to the *C*_3*v*_ point group, while the latter belongs to *T_d_*. The symmetry operations which make up a point group define the group. There are many ways to form a group, but the collection of symmetry operations is unique, i.e., there is just one specific combination.

The spectroscopic analyses which were conducted in parallel if not immediately after X-ray diffraction were those of infrared (IR) and Raman, and “UV-Vis” of aqueous solutions. Infrared (IR) and Raman spectroscopies are complementary techniques to study molecular vibrations of molecules.

### 5.1. Normal Modes and Fundamental Bands

“Normal modes” are used to depict the different vibrational motions in molecules. A normal mode is a collective, independent, synchronous motion of atoms or groups of atoms such that, provided the extended displacements remain small and the potential is parabolic, may be excited without leading to the excitation of another normal mode. Each mode is characterized by (i) a different type of motion, (ii) quantized energy levels of a harmonic oscillator for small displacements (but anharmonicity will cause the dynamics of normal modes to be independent of each other, (iii) a certain symmetry associated with it.

A molecular vibration involves the exciting of the normal mode of vibration. The atoms all undergo their displacements at the same frequency, and all pass through an equilibrium configuration simultaneously. In IR spectroscopy, transitions between quantized vibrational energy levels of a molecule are induced by the absorption of IR radiation. In Raman spectroscopy, however, transitions occur during the scattering of incident photons by molecules. At room temperature (~18 °C) almost all molecules are in their lowest vibrational states with quantum number n = 0. For each normal mode of vibration, the most probable vibrational transition is promotion to *n* = 1. The sharp peaks and strong bands (in both IR and Raman spectra) resulting from these transitions are called “fundamental bands”. Transitions to higher excited states (e.g., from *n* = 0 to *n* = 2) result in “overtone bands” that will appear much weaker than fundamental bands in intensity. Not all vibrational transitions can be studied by both IR and Raman spectroscopy because “selection rules” dictate whether a transition is allowed or forbidden. An allowed transition may result in a strong band but for a forbidden transition, its probability of manifestation is so low that a band is unlikely to be observed. If a normal mode has an allowed IR transition, we say that it is IR active; for an allowed Raman transition, we say that it is Raman active. When spectroscopists obtain both the IR and Raman spectra of the same compound, much can be deduced of its vibrational behaviour.

FTIR is an absorption determination whereby the detector measures the absorbance of infrared radiation by the compound. Each compound will absorb different amounts of each frequency resulting in a unique spectrum. Raman spectroscopy relies on inelastic scattering of photons which probes molecular vibrations. While FTIR uses a broadband infrared source, Raman spectroscopy uses a narrow-band, monochromatic light source (typically a laser beam) in order to excite the vibrations of the molecule of interest. Molecules with (functional) groups that have strong dipoles display strong peaks in the IR, while functional groups that have weak dipoles and readily undergo a change in polarizability display strong peaks in the Raman. Polarizability of a molecule refers to the ease with which electrons are distorted from their original position. The polarizability of a molecule decreases with increasing electron density, increasing bond strength and decreasing bond length.

Heisenberg’s Uncertainty Principle argues that all the constituent atoms in a molecule are in constant motion otherwise, contrary to its fundamental premise, it would be possible to determine their momenta and positions, precisely and simultaneously. Molecules exhibit three types of motions: translations, rotations and internal vibrations. A diatomic molecule such as O_2_ contains only a single vibrational motion along its bond axis. Polyatomic molecules exhibit more complex vibrations, which are actually the superposition of relatively simple vibrations, i.e., the normal modes of vibration introduced above.

### 5.2. Number of Vibrational Modes

It is easy to calculate the expected number of normal modes for a molecule made up of N constituent atoms, as follows. The “degrees of freedom” is the number of variables required to depict the dynamics of particles (and of rigid bodies in Newtonian mechanics). For an atom moving in space in three orthogonal directions (i.e., 90° to each other), three coordinates are adequate, e.g., the Cartesian coordinates *x*, *y*, *z*. There are three degrees of freedom, and its motion is restricted to that of the translational. For a molecule with N constituent atoms, their motion in three dimensions produces 3 × N degrees of freedom (since each atom has 3 degrees of freedom) which in turn form the basis for construction of 3N symmetry adapted functions. Since there are 3 × N possible displacement coordinates for a molecule, these have been expressed in Group Theory as **Г_3N_**, spoken as “gamma 3N”, which is the representation of the point group based on the 3N vectors as the basis set. In the case of the Fe**^VI^**O_4_^2−^ ion, the number of atoms N = 5, **Г_3N_** = 3 × 5 = 15. Furthermore, since the atoms are now bonded together chemically, the entire molecular ion is capable of whole-body rotation (as a single moiety) about each of the 3 axes, and translational motion along each axis, making 6 motions altogether, therefore **Г_T+R_** = 6 (the subscripts “T” and “R” stands for translation and rotation). The number of modes left for vibration is **Г_vib_** = **Г_3N_** − **Г_T+R_ =** 3N − 6 = 15 − 6 = 9. Of the 3N–6 vibrational modes in molecules (and 3N − 5 modes in linear molecules), N − 1 modes are valence (or stretching) vibrations, i.e., movement occurs along the bonds between the atoms concerned. Associated are the 2N − 5 bending vibrations in both linear and non-linear molecules. In vibration spectroscopy, the term “degeneracy” is applied to multiple vibrational modes of a molecule all at the same quantized energy state, and of the same symmetry. For species such as Fe**^VI^**O_4_^2−^, SO_4_^2−^, CH_4_ and CCl_4_, the “3N − 6” calculation is the same for each species, resulting in **Г_vib_** = 9.

### 5.3. Degenerate Vibrations in Point Group T_d_

The mathematics of Group Theory makes it possible for the vibrational modes mentioned above to be grouped into degenerate sets which are then assigned “Mulliken symbols” [76]; see also [77] for symmetry symbols. For the *T_d_* Fe**^VI^**O_4_^2−^ ion isolated in Cartesian space, the set of Mulliken symbols assigned by Gonzalez & Griffith are [78]:

**Г_vib_** = A_1_ + E + (2 × F_2_) (1)

This means that there are: one non-degenerate mode (A_1_), one doubly-degenerate mode (E) and two triply-degenerate modes (F_2_). The total number of vibrational modes for the molecular system = 3N − 6 = 1 + (1 × 2) + (2 × 3) = 9, as determined previously. (In the literature of vibration spectroscopy “F” replaces “T” in the textbooks of Group Theory). The non-degenerate mode A_1_ is a “totally symmetric” vibration and is that of the symmetric stretching of the Fe-O bond with frequency *ν*_1_ cm^−1^. All the oxygen atoms have displacement vectors along the direction of the iron-oxygen bond, and are equal in the scalar quantity (i.e., in magnitude). The vectors are pointed outwards from the Fe atom, which remains at rest. One of the two F_2_ modes, which we call F_2,1_ for the moment, is that of an asymmetric stretching with a frequency of *ν*_3_ cm^−1^. The frequencies of E and F_2,2_ are those of “bending” modes and are designated *ν*_2_ and *ν*_4_ cm^−1^ respectively by conventional notation. The high degree of symmetry of Fe**^VI^**O_4_^2−^ means that only two vibrational modes interact directly with infrared radiation, namely, *ν*_3_ and *ν*_4_, the ones where the iron and oxygen both move. These modes are the most likely to absorb or scatter infrared photons. (To visualize these vibrational motions, see the on-line video [79] to display the vibrational motions of the *T_d_* molecule CH_4_).

### 5.4. Infrared and Raman Activities

Group Theory is a useful tool to determine what symmetries the normal modes contain and predict if these modes are IR and/or Raman active. When the point group of the molecule and the symmetry labels for the normal modes are known, then group theory makes it easy to predict which normal modes will be IR and/or Raman active. If the symmetry label of the dipole moment of normal mode corresponds to *x*, *y* or *z* translations of a molecule, then the fundamental transition for this normal mode will be IR active, i.e., the electric dipole of incident radiation couples in resonance with a vibration which alters the electric dipole of the moiety under examination. In short, a normal mode is classified as IR active if it corresponds to a changing electric dipole moment of the molecule (therefore the motto “IR allowed transitions are also dipole allowed”). In Raman spectroscopy, however, there are two light waves to deal with, incident and egress. If a vibration behaves as if it were the product of two diploes, or more specifically, if the symmetry label of the “polarizability tensor” (a tensor is a matrix with vectors as elements) of a normal mode of vibration corresponds to products of *x*, *y* or *z* (such as *x*^2^, *y*^2^, *z*^2^, *xy*, *xz* or *yz*, or any linear combinations of these), then the fundamental transition for this normal mode will be Raman active. In short, a normal mode is Raman active if it corresponds to a changing polarizability. Clearly, the selection rules for IR and Raman spectroscopies are different.

Some generalizations can be made. If a molecular entity possesses little or no symmetry at all (and this is certainly not the case of Fe**^VI^**O_4_^2−^), then it is relatively straightforward to decide whether its vibrational modes will be Raman active or inactive. In fact, it is usually correct to assume that all its modes are Raman active. However, when a moiety has considerable symmetry it is not so easy to be certain because it is not always clear whether a change in polarizability occurs during the vibration. If it does, then a vibrational transition will be Raman active. It so happens that the totally symmetric vibrational modes are associated with the largest changes in polarizability with respect to the vibrational modulation of the bond, the Raman polarizability. Furthermore, the greater the change in vibration-modulated polarizability, the greater the Raman signal strength will be. Another rule-of-thumb in vibrational spectroscopy that is worth mentioning is that stretching bands are often located at higher frequencies than the bending ones.

In the developmental history of vibrational spectra, certain difficulties in spectral interpretation have been encountered. First, there are other uncertainties in symmetry-spectral relationships and these are embodied in the “exclusion rule”. It states that a normal mode of a molecule with a centre of symmetry cannot be both IR and Raman active, and perhaps neither. Furthermore, a band may exist but it is so weak that it cannot be clearly and distinctly observed in the spectral region. There are other complications. Couplings between vibrations are so weak that, whenever they become decoupled, then it is irrelevant whether the vibrations are related by a centre of symmetry. There will just be a single observable peak coincident in IR and Raman, and this is one of the difficulties in the way of fully resolving the spectrum.

With the Fe**^VI^**O_4_^2−^ ion, Group Theory predicts that the symmetric stretching frequencies *ν*_1_ and *ν*_2_ modes will not be IR active, but all four modes *ν*_1_ to *ν*_4_ are Raman active (recall that *ν*_3_ and *ν*_4_ are the two triply-degenerate vibrations), the implicit assumption is that the ferrate(VI) ion is isolated in space in the absence of any other force field external to itself. Moreover, Raman bands may be distinguished further in terms of their relative intensities measured by a polarizing filter, first parallel and then perpendicular, to the polarization of the incident radiation. If the polarization of the scattered beam is the same as that of the incident beam (intense only in the parallel direction), then the Raman line is said to be “polarized”. However, if the scattered light is intense in both the parallel and perpendicular directions, then the Raman line is “depolarized”. Only totally symmetric vibrations give rise to polarized lines. In the Fe**^VI^**O_4_^2−^ ion, only the A_1_ stretch is the polarized band, the other three bands, namely, E + 2F_2_, are depolarized. In Raman spectroscopy, the rule-of-thumb is that polarized bands can only originate from an A_1_ vibrational mode.

Readers who are interested in using the “reduction formula” to assign the spectra of ferrate(VI) from the “character table” of the *T_d_* point group will do well by following through the arithmetic set out clearly in step-by-step fashion for the case of CCl_4_ by Walton [80], then tackle the case for CH_4_ in the published MSc. dissertation of Papanastasopoulos [81]. Not mentioned by Walton [80] is the effect of the isotopic masses of chlorine (^35^Cl, ^36^Cl, ^37^Cl) which give rise to five clearly distinguishable A_1_ Raman peaks *ν*_1a_ to *ν*_1e_ in the range 462.3 to 450 cm^−1^ [82], but no such study was reported on the effect of the isotopic masses of oxygen (^16^O, ^17^O, ^18^O) on the Raman expression of ferrate(VI). This is an obvious gap in knowledge.

### 5.5. History of Analysis of Ferrate (VI) by Vibration Spectroscopy

The first wave of investigations into tetraoxo anions with the central metal ion in a high and/or highest oxidation state commenced in the 1950s to the early 1970s and laid the foundation for further research up to the present day. For the Fe**^VI^**O_4_^2−^ ion, the accompanying cation in a crystal or aqueous solution can be Na^+^, K^+^, Ca^2+^, Ba^2+^, Cs^2+^, Sr^2+^ and NH_4_^+^. An earlier paper by Becarud & Dural in 1963 described the properties and reactions of some of these ferrates [83].

In the 1960s, much effort was spent in obtaining the IR and Raman spectra of compounds containing the Fe**^VI^**O_4_^2−^ ion, in crystals and aqueous solutions, and inferences were made of their point group symmetries. During that time, the vibrational modes of ferrate(VI), namely, *ν*_1_ (A_1_ mode, iron-oxygen bond symmetric stretching), *ν*_2_ (E mode, deformational angular “bending”), *ν*_3_ (F_2_ mode, iron-oxygen bond asymmetric stretching) and *ν*_4_ (F_2_ mode, deformational “bending”) had been speculated. As already mentioned above, the *ν*_1_ and *ν*_2_ modes are not expected to be IR active. Then, in 1964, contrary to theoretical prediction, Tarte & Nizet [84] reported the appearance of a *ν*_1_ band in their IR spectra of solid K_2_FeO_4_, but no feature on the spectrum can be clearly and unequivocally ascribed to a *ν*_2_ band. Tarte & Nizet [84] labelled their peaks as follows: *ν*_1_ = 782 cm^−1^, *ν*_3_ = 809 cm^−1^ with a small shoulder at 825 cm^−1^, *ν*_4_ = 340 and 322 cm^−1^ (a doublet). Woodward & Roberts [85] conjectured that *ν*_4_ and *ν*_2_ can be so close that they can coincide, a situation also suspected by Griffith [44]. Moreover, in 1966, Griffith [44] also witnessed the frequency *ν*_1_ = 779 cm^−1^, *ν*_3_ = 827, 810 and 796 cm^−1^ (a triplet, which implies that the anionic tetraoxo entity which exists in the solid state can be a tetrahedron which has been elongated, so suggested Maghraoui et al. [86]); *ν*_4_ = 339 and 320 cm^−1^ (the comment by Woodward & Roberts [85] above for a possible hidden *ν*_2_ band also applies here), for K_2_FeO_4_. These two sets of data are tabulated in the first two columns of Table 1. In 1972, Gonzalez & Griffith [78] produced the Raman spectra of an alkaline solution of K_2_FeO_4_ and bands of all the four modes *ν*_1_ to *ν*_4_ made their appearances, Table 2. However, from considerations of the state of polarization of these lines from the Raman spectra, they decided that it was only too appropriate to reverse the order of the *ν*_1_ and *ν*_3_ modes in the literature pertaining to the IR spectra of the ferrate compound in the solid state. The assignment of vibrational modes to peaks in the third column of Table 1, and that in Table 2, represents the new order. Licht et al. [23] had used the strong 810 cm^−1^ band (akin to one of Griffith’s *ν*_3_ triplets [44]) to identify solid K_2_FeO_4_ in 2001, but with no new bands reported. In the present review, all attention is focused on ferrate(VI) itself, but it is noteworthy that the presence of the Fe(VI) ion (added as a doping agent) can render interesting spectral properties to other crystals such as K_2_SO_4_ and K_2_CrO_4_, illustrating their excited state properties candidly [87]. 

In 2019, Munyengabe & Zvinowanda [4] reported new peaks at 879 and 700 cm^−1^ in their analysis of Na_2_FeO_4_ by FTIR, but it is not known whether the presence of CO_3_^2−^/HCO^3−^ (which caused the 1920 and 900 cm^−1^ peaks in their FTIR spectrum) due to dissolved CO_2_ in air had shifted the positions of peaks reported in literature; the 770 cm^−1^ peak, however, is recognizable in reference to the *ν*_1_ band reported by Griffith [63]. Audette & Quail [88] had already cautioned against CO_2_ contamination due to the highly alkaline environment required during ferrate(VI) synthesis, as early as 1972.

In 1972, Audette & Quail [88] reproduced the IR spectra of solid K_2_FeO_4_ obtained by Tarte & Nizet [84], together with the ferrates of barium, rubidium and caesium, and all these spectra suggested deviation from *T_d_* symmetry. Independently, Griffith [44] had assigned a point group of lower symmetry than *T_d_*, namely *C_s_*, to be the “site” symmetry of the ferrate ion in the solid phase of K_2_FeO_4_, thereby acknowledging the existence of a distorted tetrahedron and that the manifestation of the *ν*_1_ band is allowed in *C_s_* albeit not in *T_d_*. To date, no crystallographer or spectroscopist has disagreed with Griffith’s downgrading of ferrate’s symmetry from *T_d_* to *C_s_* [44]. In fact, both of the Tarte & Nizet [84] and Audette & Quail [88] duets (whose works pre-dated and post-dated the 1966 publication of Griffith [63] respectively) attempted to offer plausible explanations of the deviation from perfect tetrahedral architecture; the former group suggested “asymmetric coupling” of *ν*_1_ vibrations with anions in close vicinity as a mechanism, while the latter group illustrated the degree of distortion as a function of the identity of the metal ion with Ba^2+^ ions causing the severest distortion: Ba^2+^ > K^+^ > Rb^+^ > Cs^+^. The compound Cs_2_FeO_4_ evinced the weakest *ν*_1_ peak with the least splitting of the *ν*_3_ and *ν*_4_ peaks in IR spectroscopy, which Audette & Quail [88] used as criterion to indicate the lowest degree of distortion.

A report well worth reading by chemical analysts is that of the destruction of estrogenic hormones in wastewaters by Fe(IV), Fe(V) and Fe(VI). In 2016, Karolína Machalová Šišková et al. managed to follow the pathway of the oxidation of these hormones by a combination of UV-Vis, Raman and Mössbauer spectroscopies in great detail [34]. The various spectra can be seen in the “Supplementary Information” of their paper and the Raman spectra of ferrate are uniquely different; it is an interesting scenario which lends itself to further investigation. 

### 5.6. T_d_ Symmetry of FeO_4_^2−^ Preserved under Highly Alkaline Conditions

Sharma et al. [89] reported the pK_3_ of the ferrate (VI) moiety to be 7.3. At pH = 14, the predominant Fe(VI) species will be the fully deprotonated Fe**^VI^**O_4_^2−^ and this will downplay the significance of a hypothetical reaction such as:

[Fe**^VI^**O_4_]^2−^ + **H**^+^OH^−^ ⇌ [Fe**^VI^**O_3_(O**H)**]^−^ + OH^−^ (2)

However, Goff & Murmann [45] discovered that approximately 5% of all [Fe**^VI^**O_4_]^2−^ ions in solution (5% of 5 mM) exchanged their oxygen atoms with oxygen-18 enriched water ^18^OH_2_, in the pH region 9.6 to 14, therefore:

[Fe**^VI^**O_4_]^2−^ + **^18^O**H_2_ ⇌ [Fe**^VI^**O_3_(**^18^O**)]^2−^ + H_2_O (3)

The oxygen-exchange reaction, however, does not alter the *T_d_* symmetries of the ferrate (VI) ion.

Moreover, in light of the recent work of Havenith-Newen et al. [90] which involved the probing of the hydration sphere of both cations and anions in aqueous solutions by THz spectroscopy, it is not unreasonable to suspect that chemical reactions between Fe**^VI^**O_4_^2−^ and water molecules which constitute the hydration sphere may occur. Schreyer & Ockerman [91] reported the reaction between ferrate(VI) and water, therefore:

4 Fe**^VI^**O_4_^2−^ + 4 H_2_O **→** 2 Fe_2_O_3_ (hydrated) + 3 O_2_ (g)↑ + 8 OH^−^ (4)

This reaction will certainly decrease the absorbance at the broad 505 to 510 nm band, and any spectroscopy scrutiny must be performed at high alkalinity to keep ferrate (VI) stable enough and long enough to be meaningful.

Other reactions such as addition exist between tetraoxo anions and hydroxyl ions. Carrington & Symons [92] reported the addition of hydroxyl ions to tetraoxo ions of high oxidation state metals in alkaline solutions, therefore:

Ru**^VII^**O_4_^−^ + 2 OH^−^ ⇌ [Ru**^VII^**O_4_(OH)_2_]^3−^ (unstable intermediate) (5)

Re**^VII^**O_4_^−^ + 2 OH^−^ ⇌ [Re**^VII^**O_4_(OH)_2_]^3−^ (unstable intermediate) (6)

Griffith’s own experience with osmium tetroxide [93] is such that:

Os**^VIII^**O_4_ + 2 OH^−^ ⇌ [Os**^VIII^**O_4_(OH)_2_]^2−^ (7)

The structures of both RuO_4_ and OsO_4_ in their ^1^A_1_ spin-singlet ground state are of *T_d_* symmetry, but the *T_d_* symmetry of OsO_4_ is converted to *D*_4*h*_ subsequent to the addition reaction.

Is addition of OH^−^ ions onto FeO_4_^2−^ in alkaline solution a possibility? Griffith [44] set out to discover, first by obtaining the Raman spectrum of Fe**^VI^**O_4_^2−^ in a concentrated solution of Na^+^OD^−^ in D_2_O. Another Raman spectrum is obtained in the absence of NaOD. The two spectra were identical, and it can be concluded that addition of OH^−^ ions to the Fe**^VI^**O_4_^2−^ moiety did not take place. Had addition of OH^−^ occurred, the *T_d_* symmetry would have been perturbed resulting in the shifting of the *ν*_1_ and *ν*_3_ frequencies, as in the case of osmium tetroxide [93]. Moreover, protonation of the oxygen atoms of ferrate as pH drops will no doubt alter the frequency of vibrations. Recently in 2020, Luo et al. pictorially depicted these protonated species very well [94].

To place the study of ferrate(VI) in the context of the exploration of transition metal-oxo complexes in the period up to 1970, see the review by Griffith [95]. A great number of such compounds were synthesized, analysed, examined and classified by colossal industriousness and their chemistries are the basis of many topics of interest to this day, including environmental applications. Four decades hence the publication of Griffith’s pioneering work on IR spectra of tetrahedral complexes of the transition metals, Maghraoui et al. [86] still cited Griffith’s work [44], propounding his results and implications with clarity. No work with a third type of vibrational spectroscopy, namely, “Inelastic Neutron Scattering” has been done on ferrate(VI) and this technique can perhaps resolve the symmetry issue. (See Section 7: Further Work).

### 5.7. The Search for the “FeO_4_” Molecule: Implications for Modelling [Fe^VI^O_4_]^2−^

In 1926, D.K. Goralevich conjectured that “iron tetroxide”, a discrete and electrically neutral molecular entity of empirical formula “FeO_4_”, was probably formed as a volatile and unstable compound by the reaction between BaFeO_5_ (“barium perferrate”) and an excess of dilute H_2_SO_4_ [96]. Another possible product from the reaction was thought to be H_2_FeO_5_ (“perferric acid”). “Iron tetroxide” (also known in older literature as “perferric anhydride”), should not be confused with “tri-iron tetroxide”, better known as magnetite Fe_3_O_4_; neither is it the Fe**^VI^**O_4_^2−^ ion. Goralevich also claimed to have synthesized the bright green K_2_FeO_5_·*n*H_2_O (“potassium perferrate”) by fusing ferric oxide with potassium hydroxide and an excess of potassium nitrate or chlorate. It was documented, when heated, hammered or reacted with concentrated H_2_SO_4_, “potassium perferrate” exploded! Goralevich’s work is probably one of the earliest in the literature in which the +8 oxidation state of iron is implied, although the search for the +8 state was probably not Goralevich’s intention. For enthusiasts of chemical history, Goralevich’s experience with this synthesis needs re-enacting, without a doubt. First of all, it would be useful to know how BaFeO_5_ was obtained as a starting material. Then, the procedure of synthesis laid out by Goralevich must be repeated and all products analysed by the many sophisticated instrumental methods now available. The analytical chemist will wonder what values of chemical shift of alleged Fe(VIII) compounds Mössbauer spectroscopy will show. Using Density Function Theory (DFT) calculations, Poleshchuk et al. estimated the chemical shift of “Fe**^VIII^**O_4_” to be δ = −1.40 mm s^−1^ (with respect to α-iron) and that the Fe-O bond was calculated to be 1.586 Å, assuming all Fe-O bonds are identical [97]. Sharma & Zboril (2015) produced a linear correlation equation between chemical shift and oxidations state of iron [98], therefore:

δ (mm s^−1^) = 1.084 − 0.326 × (oxidation state) (8)

Accordingly, when the oxidation state is +8, δ = 1.084 − (0.326 × 8) = −1.524 mm s^−1^. An earlier estimation by Kopelev et al. gave δ = −1.36 mm s^−1^ [99]. Therefore, if a measured chemical shift of a real iron compound is within the range 1.36 ≤ δ ≤ 1.524, it may just be possible that it is a Fe(VIII) species. Clearly, only facilities built especially for detonation research are equipped to conduct experiments involving explosions; most university laboratories are not.

By the 1960s, chemists were still not sure that “FeO_4_” had ever been prepared in any sufficient quantity to be analysed, or if it could be made at all. In the meantime, despite the concept of oxidation states being a purely hypothetical construct, the enthusiasm for the quest for Fe(VIII) has never waned. Indeed, by 1965, ruthenium and osmium had already demonstrated their highest oxidation states to be +8 in the stable oxides RuO_4_ and OsO_4_. Their spectral and other properties were characterized ([92,93]) and the two oxides have found many applications. At this juncture, in 1963, W.E. Dasent managed to publish a paper entitled “Non-existent Compounds” and also a book by the same name in 1965, in which “FeO_4_” received an honourable mention [100,101]. The book was reviewed by Schmidbaur immediately [102], who likes to opine about chemical compounds of unknown structures once in a while [103].

Theoretical chemists took a totally different approach to tackle this existential problem of “FeO_4_” by attempting to predict, in the first instance, the electronic structure of the “molecule”, often in juxtaposition with the electronic structures and geometries of existing species such as RuO_4_ and OsO_4_. In the course of these endeavours up till 2018, low-spin species such as [Fe**^VIII^**O_4_] (of *T_d_* symmetry) and [Fe**^VI^**O_2_(O_2_)] (of *C*_2*v*_ symmetry) were proposed for “iron tetroxide”; high-spin molecules included Fe(II to V) species [103]. The di-oxygen designation “O_2_” in the molecular formula represents the peroxido group O_2_^2−^ and also the superoxido free radical [(O_2_)**^.^**]^−^ (i.e., a species with a single negative charge).

In 2016, Professor W.H. Eugen Schwarz of the University of Siegen (Germany) and colleagues in China reviewed the computational chemistry regarding the metal tetroxides (MO_4_) of Fe, Ru, Os, Hs, Sm and Pu [104]. The case for iron represented by [FeO_4_]^0^ is said to be the most “delicate”, since the quantum calculations resulted in “many geometric and electronic isomers” (which no doubt makes it difficult to distinguish the most probable from the merely plausible). These workers cited half a dozen publications to make the point that computational approaches deployed so far to explain infrared and photoelectron spectra had painted “rather inconsistent pictures”. They commented, “Ab initio single-reference approaches including common density functional approximations and wave function approximations from Hartree−Fock SCF to MP2 to CCSD appear as not reliable enough, providing a variety of energy orders of the set of near-degenerate Fe·4O isomers”. Simply put, the free energy minimization calculations (subjected to constraints on input data) have produced numbers too close to call a difference. In their work, vibration frequencies were also compiled but there were no experimental data to compare with. They continued by saying that only a “highly correlated ab initio multiconfiguration approach gave sound support for the lowest isomer being a ferryl peroxide, ^1^A_1_*C*_2_*_v_*-[Fe**^VI^**(d^2^)O_2_]^2+^(η^2^-O_2_)^2^^−^ [104]”. (Note that the preference of the Fe**^VI^**(d^2^) ^1^A_1_ singlet state complex is owed to the reduced *C_2__v_* symmetry.) Practitioners of DFT will do well in taking note that, for molecular systems such as MO_4_, single reference methods such as DFT may not be ideal, and that multi-reference methods are very difficult to apply to these systems. Also, in the cases of Ru(VIII) and Os(VIII) (and especially the latter), all sorts of interesting relativistic effects come into play which stabilizes them.

A little earlier, W.H. Eugen Schwarz had already written an excellent paper (of tremendous educational value) [105] which updated the status quo of theoretical understanding of the electronic structures of the transition elements against a backdrop of a century of hard experimental data, up to the publication date of 2010. (See also the work of Philipsen & Baerends on the calculation of cohesive energies of 3*d* transition elements by Density Function Theory DFT [106]). There is certainly no lack of understanding of the electronic configuration of the elemental atoms. The valence electrons of Ru and Os are well shielded from nuclei attraction by electrons in the main shell with principal quantum number *n* = 4 (osmium’s *n* = 4 shell is full), the energy requirement to attain a specific (higher) oxidation state will not be as demanding as that of an equivalent oxidation state in Fe. Here, the concept of electrostatic attraction and repulsion in classical physics which can lead to different thermodynamic scenarios of redox reactions between the elements is not easy to dispute, but is it as easy to model mathematically?

In a publication with the interesting title “How much can Density Functional Approximations (DFA) fail? The Extreme Case of the FeO_4_ Species” [107], Schwarz’s research group answered its own question by a forthright expression in the core sentiment of their paper, “The disputed existence of oxidation state Fe(VIII) is discussed for isolated FeO_4_ molecules. Density functional theory (DFT) at various approximation levels of local and gradient approaches, Hartree-Fock exchange and meta hybrids, range dependent, DFT−D and DFT+U models do not perform better for the relative stabilities of the geometric and electronic Fe·4O isomers than within 1−5 eV.” And that is the crux of the problem with any modern theory attempting to decipher the spectra of ferrate(VI) samples. The [Fe**^VI^**O_4_]^2−^ ion does not exist in isolation. Ferrate(VI) ions are constituents of crystals together with metal cations such as Na^+^, Rb^+^, Ba^2+^ and Sr^2+^, and the NH_4_^+^ ion. In aqueous solutions, [Fe**^VI^**O_4_]^2−^ ions are surrounded by water, with propensity to react chemically [90]. The spectra obtained are properties of these physical-chemical systems in their entirety. Any hypothesis which neglects the interactions of ferrate(VI) ions with their immediate environments is bound to be inadequate and unsatisfactory.

Schwarz’s comment is followed by a positive mission statement which is most welcome, “The Fe·4O isomeric species are an excellent testing and validation ground for the development of density functional and wave function methods for strongly correlated multireference states, which do not seem to always follow chemical intuition [107]”. In full support of this vision, the authors of the present review can suggest a couple of transition metal compounds containing oxygen atoms which may serve as testing ground for computational methodologies. For example, it would be useful to know the oxidation state of Cr, the molecular architecture, the Gibb’s free energy of formation and the symmetry of a series of Cr compounds in their ground states with empirical formulae (M**^I^**)Cr_3_O_8_ and (M**^I^**)_3_CrO_8_ when a highly correlated ab initio multi-configurational approach is adopted to create virtual isomers for these compounds, and interpreted. (M is any Group I alkali metal). There is a simpler problem to solve involving just three atoms. The oxidation state of titanium in TiO_2_ which has so far been recognized as +4 has been disputed recently [108], and perhaps advanced quantum calculations can elucidate whether a computer simulated species of “titanium dioxide” in the +4 state has the lowest ground state energy of all feasible isomers.

Two years after the publication of the unreserved critiques of “Density Function Approximations” described above ([104,107]), Scharwz’s research group investigated the possibility of exhibition of the +10 oxidation state of platinum in a [PtO_4_]^2+^ isomer via a theoretical-experimental project, but they seemed to have conceded that an oxidation state of +8 is the maximum that can ever be attained by an element in any stable chemical species under ambient conditions. (Efforts to synthesize [Ir**^IX^**O_4_]^+^ did not come to fruition.) Does it mean, therefore, that the pinnacle oxidation state of +8 has already been reached in Ru and Os more than half a century ago? In a moment reminiscent of retrospection and humour, Scharwz et al. pondered whether there are further techniques to attain yet higher oxidation states, and reported that the IUPAC “algorithm” for the estimation of oxidation states is akin the following exercise: “Draw a reasonable Lewis (resonance) structure with one and two-center electron pairs, then assign the two-center pairs as localized at the more electronegative atoms” [109]. If that is the earnest recommendation of IUPAC, so be it. However, Professor Evert Jan Baerends (Vrije University Amsterdam, Holland) told a very touching story about Professor Gilbert Newton Lewis (1875–1946), exonerating “much of his thinking, for instance his generalization of the concepts of acidity and basicity, to donation and acceptance of electron pairs”, as being “fully vindicated by quantum chemistry [110]”. (Lewis was nominated for the Nobel Prize for chemistry 41 times but never won it).

Professor W.H. Eugen Schwarz was not the only theoretician to highlight problems encountered in computational chemistry. In fact, his contemporary Professor Tom Ziegler at the University of Calgary (Canada) had expressed similar sentiments in 2009 in a paper, with the title expressed as a question: “Is charge transfer transitions really too difficult for standard density functional or are they just a problem for time-dependent density functional theory based on a linear response approach? [111]”. Difficult? Yes, but fortunately, Ziegler et al. set out to remedy the situation by the “inclusion of higher order response terms readily affords a qualitatively correct picture even for simple functionals based on the local density approximation (and) derive a correction that can be added as a perturbation to charge transfer excitation energies calculated by standard TD-DFT (time-dependent density function theory)” [111]. It has become apparent that the success of these mathematical models in explaining the electronic configuration and predicting the reactivity of chemical species is a strong function of the degree of sophistication of the calculations, to various extents.

A chronological account of the development of theories on the electronic structures of transition metal complexes from 1950s to 2011, centred on Density Function Theory, Hartree-Fock and post Hartree-Fock methods, was published by the late Professor Tom Ziegler (1945–2015) of the University of Calgary (Canada) [112]. He compared the methods through their applications to permanganate [Mn^(+VII)^O_4_]^−^ and other tetraoxo species. Buijse & Baerends [113] studied chemical bonding in tetraoxo metal complexes by using MnO_4_^−^ as a prototype.

Professor Hubert Schmidbaur (University of München) and Professor Eugen Schwarz (Siegen University) continue to take a keen interest in the chemistry of metals in their high oxidation states. Together they have published a review paper in April 2021 on a compound called permanganyl fluoride. The story is about “a brief history of the molecule MnO_3_F and of those who cared for it [114]”, another humble yet humorous title from the duet.

## 6. Stability and Solubility of Iron Species in Aqueous Solutions

### 6.1. Considerations of Pourbaix Stability

Marcel Pourbaix was a corrosion chemist who compiled the first atlas of pH-E_h_ diagrams of the chemical elements including iron [115]. These diagrams have also been used to study geochemistry of rocks and minerals, and increasingly so, to predict the speciation of metallic and non-metallic elements in natural waters under wide-ranging redox and pH conditions [116]. There is a myriad of uses of Pourbaix diagrams and on this occasion, the authors of this work would like to comment briefly on the stability of Fe(VI) according to a published Pourbaix diagram.

**Figure 6:**
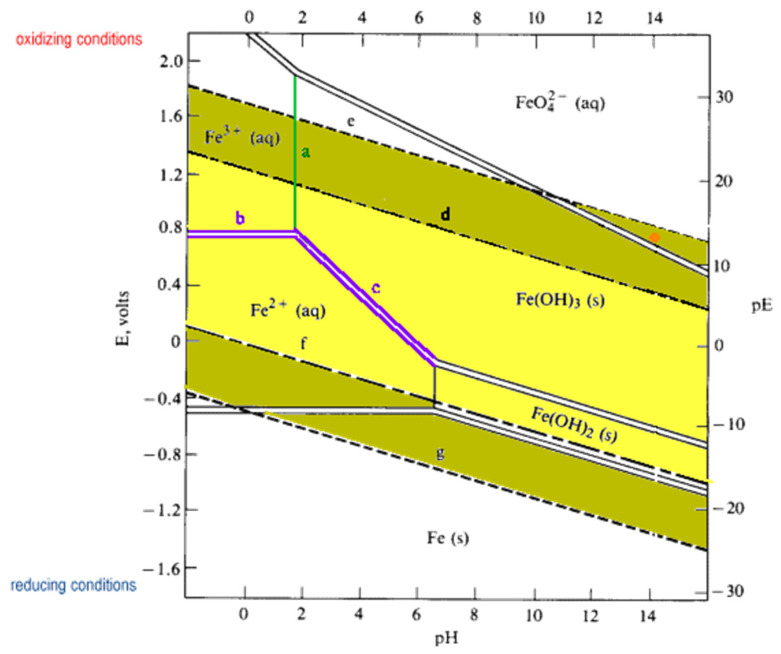
Pourbaix diagram of iron based on 1M ferrate(VI) at 25 °C [117].

A Pourbaix diagram based on the concentration of 1 mol dm^−3^ of the active species, the FeO_4_^2−^ ion in aqueous solution at 25 °C, was produced by Wulfsberg [117], Figure 6. It was adapted and discussed by at least three groups of researchers interested in Fe(VI) applications, namely, Graham et al. [118], Karim et al. [119] and Jessen et al. [120], and collectively by the Western Oregon University establishment as educational material [121].

The standard reduction potential for Fe(VI)/Fe(III) at pH = 14 is +0.71 V (Standard Hydrogen Electrode, SHE), comparable to the oxidising properties of Fe(III) in acid solution, the potential for the redox pair Fe(III)/Fe(II) being +0.77 V at pH = 0. In theory, any oxidant with a standard reduction potential at pH = 14 greater than +0.4 V is thermodynamically capable of oxidising water to oxygen (at the upper water-stability line) and water should be oxidized by any dissolved oxidizing agent, i.e., when E° > 1.23 V, see Figure 6. Likewise, in acidic solutions, at the reduction potential of E° = +2.2 V, oxidation of water by Fe(VI) is thermodynamically favourable. It also happens that the hydrolysis is kinetically rapid.

Other features on the Pourbaix diagram in Figure 6 are as follows. Line “**a**” shows the pH at which half of the iron population is Fe^3+^ and half is the precipitate Fe(OH)_2_. The solid double lines “**c**” and “**d**” separate species related by dynamic equilibria. Species in redox equilibria but not involving H^+^ or OH^−^ ions appear as horizontal boundaries, such as line “**b**”. For species involving H^+^ or OH^−^ ions they appear as diagonal boundaries because they are part of the acid-base equilibria, line “**c**”. Long-dashed lines envelope the region of stability of the water to oxidation and reduction, lines “**d**” and “**f**” respectively, while short-dashed lines fence in the practical region of stability of the water, lines “**e**” and “**g**”. Line “**d**” represents the potential of water saturated with dissolved O_2_ at 1 atm.

**Figure 7:**
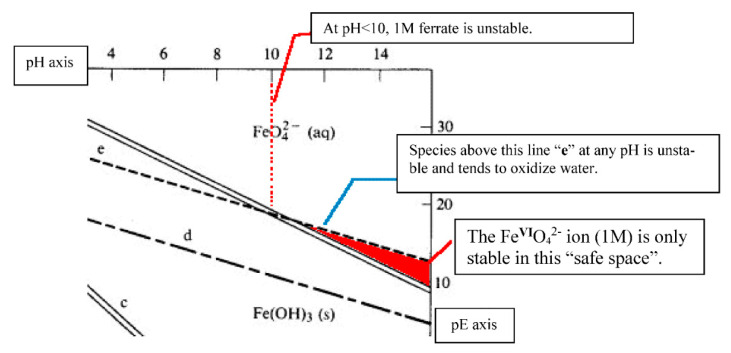
The ferrate (VI) stability region in aqueous solutions [117,121].

In practice, there are kinetic barriers to redox reactions and this is expressed in terms of an electrode ‘over-potential’ of a nominal +0.5 V [121], a voltage recognized and utilized by most industries. For the redox system Fe(VI)/Fe(III), the standard reduction potential of which is +0.71 V at pH = 14, the necessary applied potential for the oxidization of H_2_O is 0.71 + 0.5 = +1.21 V, which is not likely to be attained by the system alone. (Diagrammatically, this is moving the thermodynamic state of the solution to a vertical position north of the dotted line “**c**” at pH = 14). Immediately, it can be observed that ferrate(VI) is only stable within a small triangular region on the Pourbaix diagram formed by the FeO_4_^2−^/Fe(OH)_3_ boundary line which demarcates regions of species dominance, the portion of line “**c**” beyond pH = 10, and the vertical pE axis on the right-hand side of the Pourbaix diagram, see Figure 7.

Note that the pE scale (axis on the right-hand side of Figure 6 and Figure 7) is intended to represent the concentration of the standard reducing agent (e.g., the electron e^−^) analogously to the pH scale representing the concentration of protons H^+^. High potentials or high pE represent an oxidizing environment, while low potentials or low pE values signify reducing environments. Values of pE are obtained from reduction potentials by dividing E° by 0.059.

### 6.2. The Sacrificial Decomposition of Tetraoxoferrate(VI)

#### 6.2.1. The Quest for the Optimal pH as an Operational Parameter in Water Treatment

The kinetics and mechanisms of the decomposition of Fe**^VI^**O_4_^2−^ (aq) is an ongoing research area. The interest has been both theoretical and practical. From the point of view of potable water and wastewater treatment, it is essential to know whether the amount of applied ferrate is only of the necessary minimum, and not excessive, under the prevailing conditions. In alkaline conditions, a sludge is formed as the end product when redox is carried to completion, but the portion of Fe(VI) which ends up as Fe(III) or Fe(II) without reacting with water pollutants to the point of nullification of their potential toxic effects represents a sacrificial cost. Therefore, the amount (and therefore the rate) at which ferrate(VI) is consumed by competitive reactions must be minimized. This demands physical chemists and water engineers in collaboration to bring fruition to such an optimization project. Unreacted Fe(VI) can be determined quantitatively by its reaction with 2,2-azino-bis(3-ethylbenzothiazoline-6-sulfonate) solutions, abbreviated to “ABTS” in the literature, or potassium iodide KI solutions ([32,74]). This completes the mass balance for iron atoms.

Correlating the findings of a handful of dedicated research groups in the subject matter of ferrate(VI) decomposition will open up into a panorama of some complex chemical phenomena for readers, to help them “see the wood from the trees”.

#### 6.2.2. The Pioneer Work of Goff & Murmann

A good commencement point for discussion of the decomposition of ferrate(VI) is the pioneer work of Goff & Murmann [45]. While preparing aqueous solutions of K_2_FeO_4_ they detected the extemporaneous release of gaseous O_2_ molecules with a concomitant increase in pH of the solution, which became alkaline: Goff & Murmann chose to express their preliminary finding this way:

4 FeO_4_^2−^ + 10 H_2_O → 4 Fe^3+^ + 3 O_2_ (g)↑ + 20 OH^−^. (9)

The expression is the immediate result of the summation of two half-reactions, namely, the reduction of Fe(VI) to Fe(III) and the oxidation of water. Actually, iron(III) hydroxide will form in alkali, therefore:

4 FeO_4_^2−^ + 10 H_2_O → 4 Fe(OH)_3_ + 3 O_2_ (g)↑ + 8 OH^−^ (10)

Goff & Murmann [45] examined the isotopic abundance of ^18^O in released O_2_(g) due to the decomposition of Fe**^VI^**O_4_^2−^ ions in oxygen-18 enriched water, of pH = 0 to pH = 8.8. A general trend could be observed within this pH range. From pH = 0 to 1.48, the proportion of gaseous O_2_ which originated from H_2_O decreased from ~100% to 90%. (The only other oxygenated species present is Fe**^VI^**O_4_^2−^ and therefore at pH = 1.5 it contributed to 10% of the released oxygen). In the range of pH = 4.1 to 4.9, 73% of O_2_ came from H_2_O. In the near-neutral range of pH = 6.8 to 7.3, the fraction of O_2_ stemmed from H_2_O dropped to 59%. In the last range examined, pH = 7.3 to 8.8, only 38% (i.e., less than half) of O_2_ was derived from water, and the Law of Conservation of Mass demands that 62% of the released O_2_ arose from ferrate.

Interestingly, Goff & Murmann [45] twice mentioned a hypothetical dimeric (di-iron) species which might have played a pivotal role as an intermediate in the mechanism of decomposition of Fe**^VI^**O_4_^2−^ (aq). The di-iron species was deemed significant in the acidic regime, but was not thought to be involved when the solution is alkaline (were it to exist at high pH at all), as a graphical method used by these researchers had inferred. These researchers did not cite the origin of the idea of a di-iron species explicitly, but such a moiety was given a formula and incorporated into mathematical conceptualization of the involvement of a dimeric intermediate, by the late Professor James “Jim” David Carr (1938–2020) of the University of Nebraska-Lincoln (see below).

For these empirical observations, the absolute value of the net rate of consumption (“disappearance”) of FeO_4_^2−^ ions (which is often expressed as the differential calculus term |−d[FeO_4_^2−^]/dt| in rate equations in contemporary literature) was determined using absorbance measurements of the 505 nm peak in the visible spectrum (wavelengths of 505–515 nm are very common). No more experimental data and theoretical surmises were provided by Goff & Murmann for conditions beyond pH = 8.8, but it is clear that the data they collected so far allows more than one reaction scheme leading to ferrate(VI) decay to be posited. The data suggests the possibility that two pathways can proceed simultaneously. For example, the splitting of solvent H_2_O molecules as the causation of evolved O_2_ dwindled steadily as pH is raised but never halted abruptly, at least up till pH = 8.8 is reached, as far as the data shows. The difference in parallel pathways is perhaps the rate of propagation. These qualitative propositions have sparked subsequent research for five decades and are still the subject of discussion and debate today.

#### 6.2.3. The Role of Group II Metal Ions in Ferrate(VI) Decomposition in Alkaline Waters

In 2018, Chen et al. followed the “oxidation of water” by ferrate(VI) from pH 7 to 9 meticulously by UV-Vis spectroscopy [122], whereby the highest pH range examined by Goff & Murmann (i.e., pH = 7.3 to 8.8, [45]) was re-investigated, in essence. Akin to the work of Goff and Murmann [68], the experimentation of Chen et al. [122] also included ^18^O labelling. The provenance of both atoms of evolved O_2_ molecules was ascertained by experiments which involved dissolving K_2_[Fe(^16^O)_4_] in a mixture of H_2_(^18^O)/H_2_(^16^O), and dissolving K_2_[Fe(^18^O)_4_] in H_2_(^16^O). Their experiments demonstrated convincingly that both atoms of O_2_ originated from ferrate exclusively, i.e., the decomposition of [Fe(^18^O)_4_]^2−^ in H_2_(^16^O) produced (^18^O)_2_. The net result of the decomposition is that it is a self-decay. In comparison, Goff & Murmann [45] reported a figure of (100 − 38.7)% = 61.3% of O_2_ originating from ferrate(VI) within the same pH range. One other difference between the thinking of the two research groups is that Chen et al. [122] did not relinquish the idea of the formation of a di-iron species as part of the overall mechanism. On the contrary, they resorted to Density Function Theory (DFT) calculations to support the feasibility of the existence of such moieties. These are the steps proposed for ferrate(VI) decomposition proposed by Chen et al. [122]:

Step 1, protonation of a ferrate(VI) ion:

Fe**^VI^**O_4_^2−^ + H^+^ ⇌ [Fe**^VI^**O_3_(OH)]^−^

Step 2, condensation to form diferrate(VI) ion:

2[Fe**^VI^**O_3_(OH)]^−^ ⇌ [(Fe**^VI^**)_2_O_7_]^2−^ + H_2_O

Step 3, O-O coupling and O_2_ evolution:

[(Fe**^VI^**)_2_O_7_]^2−^ → [(Fe**^IV^**)_2_O_5_]^2^^−^ + O_2_

Step 4, reductive decomposition of Fe(IV) to Fe(III):

[(Fe**^IV^**)_2_O_5_]^2−^ + 8H^+^ → 2Fe^3+^ + ½ O_2_ + 4H_2_O.

In chemistry, the protocol of balancing half-equations of redox reactions in alkaline solutions is such that protons (hydroxonium ions) do not appear in the final equation, and one may be forgiven for writing the following equation to represent an overall stoichiometrically correct picture for Step 4, therefore:

2[(Fe**^IV^**)_2_O_5_]^2−^ + 8H_2_O → O_2_ + 4Fe**^III^**(OH)_3_ + 4OH^−^. (11)

There is an aspect of the work of Chen et al. [122] which deserves attention by water and wastewater engineers. They found that the Group II metals Mg, Ca, Sr and Ba will help release O_2_ from Fe**^VI^**O_4_^2−^, therefore:

2 [Fe**^VI^**O_4_]^2−^ + Ca^2+^ → {Ca[Fe**^VI^**O_4_]_2_}^2−^ → {Ca[Fe**^IV^**O_3_]_2_}^2−^ + O_2_ (12)

d[O_2_]/dt = *k*[M^2+^][Fe**^VI^**O_4_^2−^]^2^ (13)

The reactivity (oxidizing powers) of the calcium diferrate(IV) ion should be investigated further because natural water may be used for industrial purposes and the water may be “hard” (laden with chalk). Had the metal ferrates(IV) been proven to be more reactive than ferrate(VI), the decomposition of Fe**^VI^**O_4_^2−^ may not be sacrificial after all.

#### 6.2.4. Protonated Forms of Ferrate(VI)

**Figure 8:**
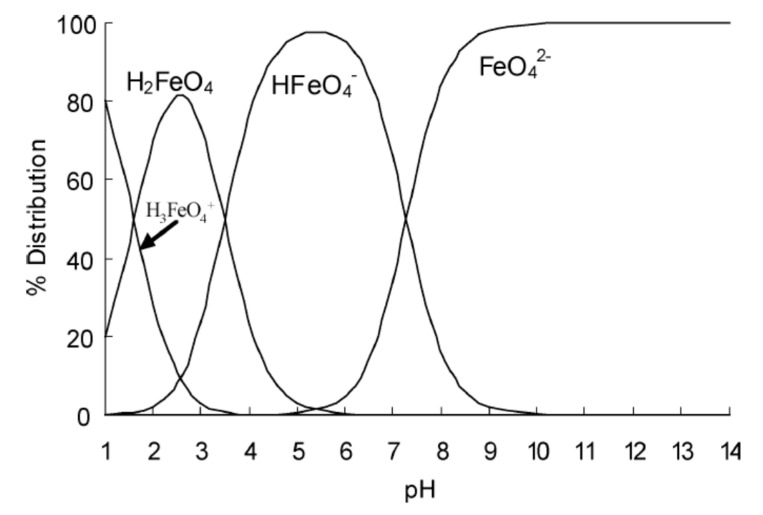
Distribution of protonated species of Fe(VI) as a function of pH [124].

For the sake of the following discourse, let the new label **P_6,0_** denote the species Fe**^VI^**O_4_^2−^, the first subscript “6” to indicate the oxidation state +6, and the second subscript “0” to keep an account of the number of hydrogen atoms present. By the same manner, **P_6,1_**, **P_6,2_** and **P_6,3_** represent the singly, doubly and triply protonated Fe(VI) ions: [Fe**^VI^**O_3_(OH)]^−^, [Fe**^VI^**O_2_(OH)_2_]^0^ and [Fe**^VI^**O(OH)_3_]^+^ respectively. The precision of measurements of the respective ionization constants of **P_6,3_** and **P_6,2_** (pK_1_ = 1.6, pK_2_ = 3.5) are not in doubt [89]. In 2001, Sharma et al. determined pK_3_ of **P_6,1_** to be 7.2 from experiments conducted in brine [89]. This value is approximately half a pH unit lower than that produced earlier by Rush et al. [123] of 7.8, in 1996. The apparent discrepancy was due to the different concentrations of buffers used, but does not affect ensuing arguments in the elucidation of mechanisms in Fe(VI) decomposition decisively. A speciation diagram for ferrate (VI) from pH = 1 to 14 was produced by Tiwari et al. [124], Figure 8.

At pH = 1.48, the closest pH value to pK_1_ = 1.6 as far as the availability of experimentally acquired data of the origin of O_2_ during the decomposition of Fe(VI) species is concerned [45], the mole ratio [**P_6,2_**] to [**P_6,3_**] can easily be calculated by the Henderson-Hasslebach equation [125]:

1.48 = 1.6 + log_10_ ([**P_6,2_**]/[**P_6,3_**]) (14)

Therefore, [**P_6,2_**]/[**P_6,3_**] = ¾. The mole fraction of **P_6,2_** is 3/(3 + 4), or 43%.

#### 6.2.5. Decomposition of Ferrate(VI) in Acidic Solutions

The research group of the late Professor Justin P. Roth (1970–2016) at Johns Hopkins University (USA) had studied the decomposition of ferrate(VI) in the strongly acidic regime pH = 1 to 3 by UV-Vis and ESR spectroscopies, in combination with ΔG minimization calculations for theoretical development of kinetics and mechanisms [126]. Their work lent practical and theoretical support to the empirical observation of Goff & Murmann [45], in the sense that the released O_2_ originated chiefly from the solvent (water molecules) when conditions are acidic; for example, that the proportion was 90% when pH = 1.48 as reported by Goff & Murmann (and stated above, [45]). The initiation step for the decomposition of ferrate(VI) is protonation of the **P_6,2_** monomer to become **P_6,3_**, followed by the dimerization reaction **P_6,3_** + **P_6,3_** and oxygen-coupling (formation of a formal chemical bond between two oxygen atoms, one from each monomer, and neither one having involved in the dimerization step). Roth’s group made the explicit assumption that “the reaction of the iron(VI) starting material is in a triplet state and forms an anti-ferromagnetically coupled μ-1,2-oxo-bridged di-iron(VI) intermediate [126].

#### 6.2.6. The Dichromate Analogy

It is plausible that the hypothesis of the formation of a di-iron complex was inspired by the established pH-dependent chromate-dichromate inter-conversion:

2[CrO_4_]^2−^ + 2H^+^ ⇌ 2[O_3_Cr-OH]^−^ ⇌ [O_3_Cr-O-CrO_3_]^2−^ + HOH. (15)

**Figure 9:**
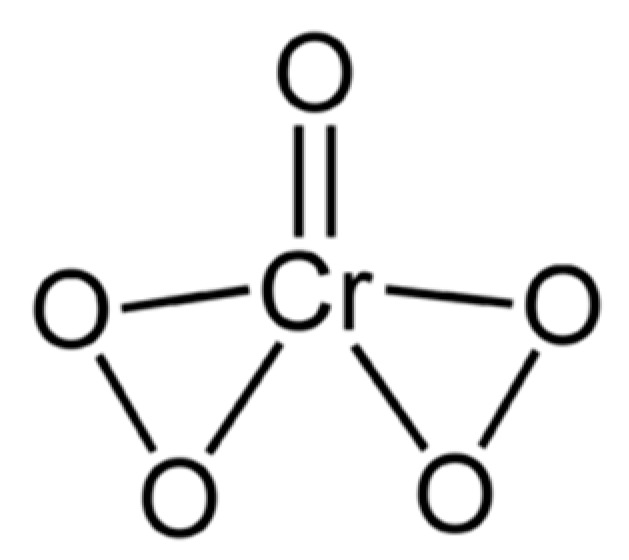
The molecular structure of chromium(VI) pentoxide.

Note that reported pK_a_ values of the monoprotic Brønsted-Lowry acid [HCrO_4_]^−^ are in the range 5.9 to 6.5 [127], but [HFeO_4_]^−^ is a much stronger acid, with pK_a_ = 1.6 [89]. Moreover, it is uncertain whether an oxo bridge (-O-O-) will form intra-molecularly within the dichromate ion itself, and with none reported, the “similarity” between diferrate and dichromate ends with sharing the same formula [(M**^VI^**)_2_O_7_]^2−^. While it is thermodynamically feasible for dichromate to split water in acidic solution (the Gibb’s free energy being ΔG = −nF(1.33 V − 1.23 V), a negative value), it does not seem to happen no matter how long one observes the aqueous system by UV-Vis spectroscopy. Dichromate seems to prefer to interact with water according to Eqt. 15 at room temperatures, via the backward reaction with rate constant *k_b_* = 4.9 × 10^−4^ M^−1^s^−1^. The overall forward reaction (formation of dichromate) is much faster, with rate constant *k_f_* = 1.8 M^−1^s^−1^, i.e., by about 3600 times [128]. There are, however, chromium(VI) peroxo complexes with side-bound O-O linkages, e.g., chromium(VI) pentoxide CrO_5_ (note that the oxidation state of chromium here is not +10), with oxygen atoms chemically bound to each other, Figure 9.

Chromium(VI) peroxide is formed by the addition of acidified H_2_O_2_ to chromates (or dichromates). The yellow chromate solution turns dark blue as CrO_5_ is formed.

CrO_4_^2−^ + 2H_2_O_2_ + 2H^+^ → CrO_5_ + 3H_2_O (16)

Chromium(VI) peroxide CrO_5_ can be extracted into diethyl ether for observation, otherwise it will oxidize any excess H_2_O_2_ present in the aqueous mixture rapidly, rendering the solution green as Cr^3+^ ions are formed, with evolution of O_2_ [129], therefore:

2CrO_5_ + 7H_2_O_2_ + 6H^+^ → 2Cr^3+^ + 10H_2_O + 7O_2_ (17)

Chemical analysts should take note that crystals of chromium(III) chloride hexahydrate [CrCl_2_(H_2_O)_4_]·Cl(H_2_O)_2_ dissolve in water to furnish a green colouration, only to turn violet after a day if unperturbed. Ligand exchange between Cl^−^ ions and H_2_O molecules on the Cr^3+^ ion is sluggish, but the process can be accelerated by warming the solution. The reverse colour change will be observed if one commences with dissolving chromium(III) sulphate in water.

Roth’s group [126] and Rush’s group [123] had pointed out that many researchers experienced difficulty in studying the reduction of Fe(VI) in solutions of extreme acidity, with concomitant oxidation of water, because the reduction Fe(VI)→Fe(III) is too fast to be monitored with precision. On the contrary, oxidation of ethanol to ethanal by dichromate in acid solution at room temperature, for example, can be observed throughout a typical time span of 15–30 min in the laboratory.

#### 6.2.7. Descriptions of Some Proposed Reaction Pathways

**Figure 10:**
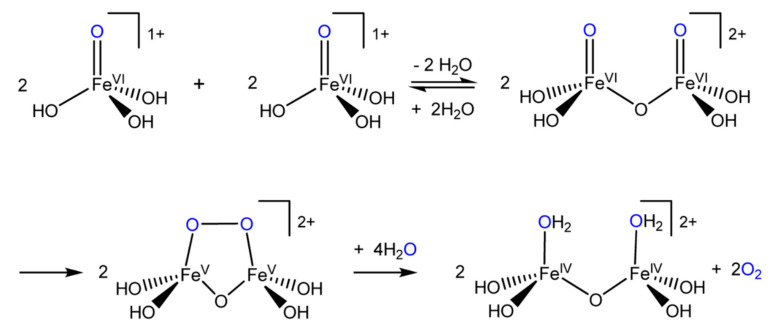
Oxidation of water molecules by Fe(V) dimeric complex [126].

**Figure 11:**
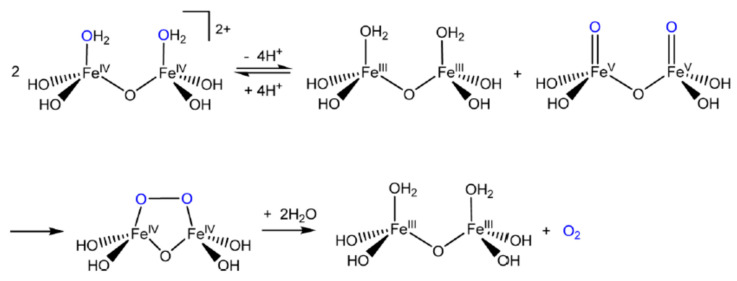
Oxidation of water molecules by Fe(IV) dimeric complex [126].

**Figure 12:**
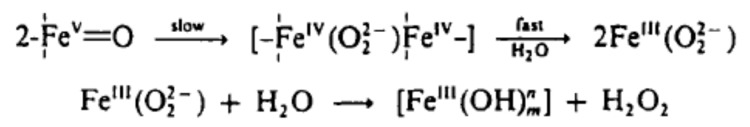
A diferrate(IV) intermediate acting as the O_2_ releasing agent [130].

Oxygen-coupling (abbreviated to “OC” in the literature) is accompanied by the reduction of both iron atoms from the +6 to +5 state. The solvent (water molecules) then enters into redox reaction with the Fe(V) dimeric complex. The central pentagonal ring of the dimer is opened by addition of hydrogen atoms (from H_2_O) onto the two oxygen atoms which formed the –O-O– bridge, thereby releasing gaseous O_2_ from H_2_O molecules. Concomitantly, Fe(V) is reduced to Fe(IV), for both iron atoms. A first order reaction for the decomposition of K_2_FeO_4_ with respect to the di-iron species H_4_(Fe^V^)_2_O_7_^2+^ was established. Finally, the Fe(IV) state is reduced to Fe(III) in the form of hydrated ferric ions [Fe(H_2_O)_6_]^3+^ in highly acidic solutions. This above is one of three scenarios for the oxidation of water proposed by Roth’s group [126], and is depicted in Figure 10. Roth’s second scheme involved the formation of Fe(II) species, but the oxo-coupled Fe(V) pentagon ring (identical to the one in Figure 10) is also formed as an intermediate; it releases O_2_ on engaging water in redox reaction, same as in Figure 10. In Roth’s third scheme portrayed in Figure 11, the pentagonal ring of the intermediate contains two Fe(IV) atoms. The Fe(IV) intermediate releases O_2_ and a diferrate(III) complex when attacked by water. Roth’s account, however, is not the first in the literature to mention a diferrate(IV) complex. In a conference held in 1989 (conference paper published in 1991), Bielski [130] described how an oxygenated Fe(V) species dimerized to become an Fe(IV) peroxo moiety, which subsequently produces Fe(III) hydroxides and H_2_O_2_ on being attacked by water, Figure 12. The Fe(II), Fe(IV) and Fe(V) intermediates encountered in Roth’s schemes are extremely short-lived and this is one factor which makes it difficult to distinguish between proposed pathways; this is noteworthy to all researchers. Nonetheless, in her report to the U.S. Department of Energy (2015) concerning fundamental research into high-valence metal catalysts for the extraction of hydrogen (from H_2_O) as fuel, Roth utilized Density Function Theory (DFT) calculations to study O-O formation in Fe(VI) species. She stated her assumptions, “Ferrate was formulated as a ground state triplet and diferrate was assumed to be in unrestricted singlet state due to strong anti-ferromagnetic coupling through the μ-oxo bridge” and that “the experimental and computational study of ferrate/diferrate reactivity provides benchmark ^18^O KIEs (kinetic isotopic effects) for various modes of O−O bond formation by synthetic and natural water oxidation catalysts [131].” In the same report, a study of water oxidation using ruthenium catalysts based on earlier work was also discussed [132].

The overall stoichiometry for the redox reaction between tetraoxoferrate(VI) ions and water molecules in acidic solution is:

4[H_3_Fe**^VI^**O_4_]^+^ + 8H_3_O^+^ → 4Fe^3+^ + 3O_2_ + 18H_2_O (18)

It is uncertain whether **P_6,2_** (a significant 43 mol% at pH = 1.48) plays a role in the initiation of a reaction scheme which leads to the decomposition of ferrate(VI), in the same manner as **P_6,3_** did (57 mol% at pH = 1.48). Nevertheless, **P_6,3_** will be replenished by the protonation of **P_6,2_** according to Le Chatelier’s principle as reduction of Fe(VI) entities proceed (**P_6,3_** ⇌ **P_6,2_** + H^+^, pK_1_ = 1.6).

In 2014, the research group of Professor Urs von Gunten (École polytechnique fédérale de Lausanne, Switzerland) and other colleagues reported that, in acidic solution, Fe**^VI^**O_4_^2−^ decomposes to Fe(III) species in both acidic and alkaline environments, but H_2_O_2_ is formed in acid while O_2_ is released in alkali [133]. Their work is a good reminder for water and wastewater engineers. They wrote in summation, “[in] the transformations of reactive ferrate(VI), perferryl(V), and ferryl(IV) to the much less reactive Fe(III), H_2_O_2_, or O_2_, the observed oxidation capacity of ferrate(VI) is typically much lower than expected from theoretical considerations (i.e., three or four electron equivalents per ferrate(VI)). This should be considered for optimizing water treatment processes using ferrate(VI) [133]”. This is, in fact, what the authors of the present review called “sacrificial decomposition of ferrate(VI)”.

#### 6.2.8. The Dichromate Analogue Incorporated into Mathematical Formulation

Note that the idea of a di-iron species as a reactive intermediate was formalized by the late Professor James David Carr (University of Nebraska-Lincoln, USA) in a conference held in 1984 (Williamsburg, VA, USA) [134]. The idea came to the foreground again in a 2008 conference, with Carr presenting a paper on the oxidation of nitrogenous compounds by ferrate(VI) in aqueous solution [135]. Note that Goff & Murmann [45] were already aware of the hypothesis of the existence of a di-iron species in 1971, but did not elaborate on its origin; they merely disputed its involvement in ferrate(VI) decomposition under alkaline conditions.

First of all, in consideration of all redox actions between ferrate(VI) and water contaminants targeted for elimination, Carr routinely took into account the rate of sacrificial decomposition of ferrate(VI) in the fashion of a professional engineer, reactions which proceed in parallel with the desired attack on water pollutants, i.e., the wastage. A “general” rate equation for the decomposition of ferrate (VI) as a result of oxidation of water was expressed as a linear combination of two terms:

|−d[Fe(VI)]/dt| = k_1_[Fe(VI)] + k_2_[Fe(VI)]^2^ (19)

This means that the rate is both 1st and 2nd order with respect to Fe(VI), hinting at complex mechanisms. The decomposition is much faster at low (acidic) pH and is observed in laboratories commonly. The 1st term in Equation (19) can be resolved further:

k_1_[Fe(VI)] = |k_(6,2)_[**P_6,2_**] + k_(6,1)_[**P_6,1_**] + k_(6,0)_ [**P_6,0_**]| (20)

Carr’s arrival at an expression for the 2nd order term was interesting and it involved the quadratic independent variable [Fe(VI)]^2^. (In fact, Equation (19) is a quadratic equation y = ax^2^ + bx + c, with c = 0 in this case). Carr was searching the best way to express, mathematically, [Fe(VI)]^2^ in terms of the concentration of the simplest known Fe(VI) moiety, namely [**P_6,0_**]^2^. Carr resorted to an algebraic expression in the physical chemistry of chromium, that of the equilibrium constant of the inter-conversion of chromate and dichromate in aqueous solution:

2CrO_4_^2–^ + 2H^+^ ⇌ Cr_2_O_7_^2–^ + H_2_O (21)

The equilibrium constant is:

K_eqm_ = [Cr_2_O_7_^2−^]/([CrO_4_^2−^]^2^ × [H^+^]^2^) (22)

The numerical value of K_eqm_ is of the order ~10^14^ according to Carr (without citing the source for the claim), but Masterton & Hurley (2016) gave a (numerical) value of 3 × 10^14^ (mol/litre)^−3^ [136]. However, a spectrophotometric determination by Smith & Metz [137] gave a much higher value of 1.3 × 10^16^ (mol/litre)^−3^. Brito et al. [138] showed that protonation of chromate precedes dimerization, therefore:

2 [HCrO_4_]^−^ ⇌ Cr_2_O_7_^2−^ + H_2_O (log_10_K_eqm_ = 2.2). (23)

Note that Steps 1 and 2 proposed by Chen et al. (as above, [122]) followed the same analogy with the chromate-dichromate inter-conversion in essence. In any case, Carr hypothesized the existence of the “diferrate” Fe_2_O_7_^2−^ ion as a congener of Cr_2_O_7_^2−^ (dichromate) as far as redox reactions are concerned, since both are powerful oxidants, and both depend on protonation of the monomer to form the dimer. The hard question is, mole-for-mole, to what extent will diferrate(VI) and dichromate(VI) oxidize an equilmolar of refractory toxic compound in aqueous solution under the same conditions of pH and temperature, and ionic strength? More importantly for this particular exercise in mechanism elucidation, the pH-dependence of concentration of the more active oxidizing species (Le Chatelier’s Principle in action) suspected to be the “diferrate” has to be addressed. Analogous to the chromate-dichromate equilibrium, the formation constant for the “diferrate” species can be written as:

K’_eqm_ = [Fe_2_O_7_^2−^]/([FeO_4_^2−^]^2^ × [H^+^]^2^) (24)

The above expression can be re-arranged to give:

[FeO_4_^2−^]^2^ = (K’_eqm_ × [H^+^]^2^)^−1^ × [Fe_2_O_7_^2−^]

Multiplying both sides of the equation by k_2_,

k_2_ × [FeO_4_^2−^]^2^ = [k_2_ × (K’_eqm_ × [H^+^]^2^)^−1^] × [Fe_2_O_7_^2−^].

Carr then grouped the multiplicative product [k_2_ × (K’_eqm_ × [H^+^]^2^)^−1^] on the right-hand side of the equation into a single factor called k_D_, so that:

k_2_[FeO_4_^2−^]^2^ = k_D_[Fe_2_O_7_^2−^].

Substituting the expression for [Fe_2_O_7_^2−^] from Equation (24),

k_2_[FeO_4_^2−^]^2^ = k_D_ × K’_eqm_ × [H^+^]^2^ × [FeO_4_^2−^]^2^.

Cancelling [FeO_4_^2−^]^2^ on both sides of the equation,

k_2_ = k_D_ × K’_eqm_ × [H^+^]^2^;

then taking logarithms,

log_10_k_2_ = −2(pH) + log_10_(k_D_. K’_eqm_) (25)

Equation (25) is an equation of a straight line. Carr then plotted log_10_k_2_ vs. pH using a numerical value of K’_eqm_ = 1 × 10^14^ and obtained a straight line graph with a negative slope, and a linear correlation coefficient 0.99. Such is the humble yet efficacious beginning of the conceptualization of a di-iron oxidizing agent. The etymology of the name “diferrate” is hardly alluded to within the literature post-2010. Roth’s group should be given credit for citing Carr’s work and alluding to the dichromate connection [126]. 

However, note that Equation (12) does not contain a term involving **P_6,3_** and is therefore not applicable to the model proposed by Roth’s group [126]. It is likely that Carr’s study [135] only focused around pH values close to pK_2_ = 3.5, and near-neutral pH values close to pK_3_ = 7.2. So, why not include studies at pH = pK_1_ = 1.6 to complete the acidic-to-neutral range? The work of Professor Urs von Gunten et al. [133] may furnish a clue. Their work examines the formation of dimers between Fe(VI) species and the determination of the second-order rate constants of these dimerizations. The pH range studied was 1 to 8.2, but stated that the most consistent results laid in the range 3 ≤ pH ≤ 8, with no further allusion to the quality of data obtained from pH = 1 to 3. Why it is so difficult to obtain precise and consistent data in this range of acidic pH? It is possible that the reactions are so rapid as to defy monitoring by conventional laboratory instrumental analysis, as Roth’s group [126] and Rush’s group [123] had alluded to.

#### 6.2.9. Strong Dependency of Ferrate(VI) Decomposition on pH Observed in Laboratories

**Figure 13:**
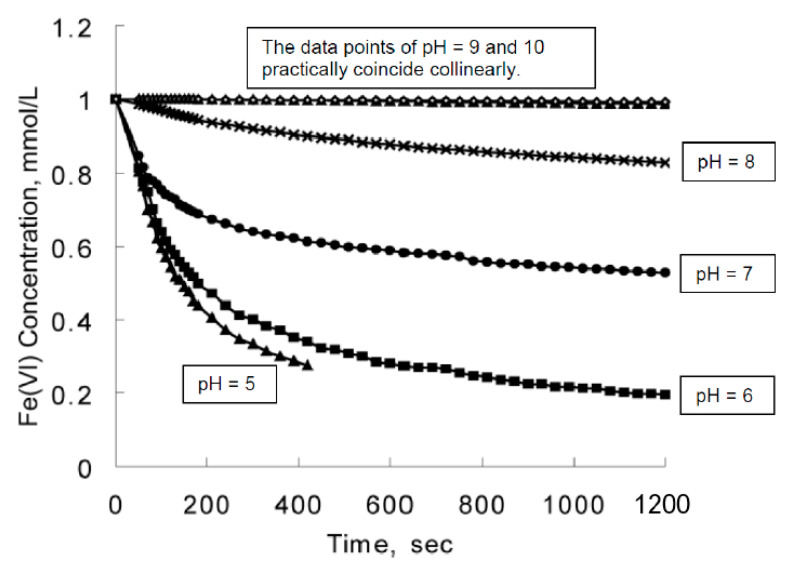
Rate of decomposition of ferrate(VI) as a function of pH [83].

Akin to the mechanistic model of Roth et al. [126], Gunten et al. [133] had also advocated dimerization of Fe(VI) species as the initiation step, the formation of Fe(V) and Fe(IV) intermediates and H_2_O_2_ (and the chemical reaction between the iron intermediates and H_2_O_2_) as propagation steps, and the formation of Fe(OH)_3_ and O_2_ as the termination steps in the entire reaction scheme of ferrate (VI) decomposition. The following dimerizations can occur: **P_6,0_** + **P_6,1_**; **P_6,1_** + **P_6,1_**; **P_6,1_** + **P_6,2_**; **P_6,2_** + **P_6,2_**; **P_6,2_** + **P_6,3_**; **P_6,3_** + **P_6,3_**. However, Rush & Bielski ([139,140]) asserted that 1st order kinetics is the only possibility for **P_6,1_**, **P_6,2_** and **P_6,3_** at pH < 7. The relative abundances of these Fe(VI) species are a strong function of pH (see speciation diagram in Figure 8) and therefore the overall rate of reduction of Fe(VI) to Fe(III) is heavily pH dependent. The more acidic an aqueous solution, the faster ferrate(VI) decomposes, as testified by the experiments of Tiwari et al. [124], Figure 13.

Amongst the experimental results provided by Goff & Murmann [45], there are these two data points: (i) in the region pH = 6.8 to 7.5, an average of 56% of O_2_ released originated from the water solvent; (ii) in the region pH = 7.3 to 8.3, the proportion was 43%. Therefore the contributions to gaseous O_2_ by solvent (H_2_O) and solute (Fe**^VI^**O_4_^2−^) were equal (50%:50%) within the range 7.5 < pH < 8.3, in the slightly alkaline region. Furthermore, the average amount of solvent-derived O_2_ in the region pH = 7.3 to 8.8 (an increase in alkalinity of 0.5 pH unit from 8.3) fell to 38%. Note that at pH = 8.8, the mole fraction of **P_6,1_** in the Fe(VI) population is a mere 2.5% (since pK_3_ = 7.2), the rest of the population is the unprotonated **P_6,0_** which can be hypothesized as the origin of (100% − 38% = 62%) gaseous O_2_. By a graphical method, Goff & Murmann [45] ruled out the formation of dimers and generated polemics to this day. Nevertheless, it is not difficult to posit more than one reaction scheme for the decomposition of ferrate(VI), and that one pathway becomes more significant over others as pH increases gradually; parallel pathways are always a possibility. In fact, for pH = 9 to 10. Luo et al. [94] proposed the initiation step to be one in which **P_6,0_** reacts with H_2_O rather than dimerization (**P_6,0_** + **P_6,0_**) because the former has a lower requirement of activation energy, therefore:

**P_6,0_** + H_2_O → Fe**^IV^**O_3_^2−^ + H_2_O_2_ (26)

In addition, Luo et al. [94] incorporated many reactions which were reported in the literature regarding the decomposition of **P_6,0_** together with the order of these reactions and their rate constants. All five oxidation states of iron from +6 to +2 are involved in the propagation steps. None of these reactions can be dismissed as insignificant to the overall mechanistic picture. Some include the release of O_2_ and increment of pH (either by consumption of protons or production of hydroxyl ions) concomitantly, and can be considered the termination steps. Two such reactions and their rate constants at pH = 9 are as follows [94]:

Fe**^IV^**O_3_^2−^ + H_2_O_2_ + 2 H^+^ → Fe**^II^**(OH)_2_ + O_2_ + 2 H_2_O (*k* = 1 × 10^4^ M^−1^s^−1^) (27)

HFe**^V^**O_4_^2−^ + H_2_O_2_ + H_2_O → Fe**^III^**(OH)_3_ + O_2_ + 2 OH^–^ (*k* = 4 × 10^5^ M^−1^s^−1^) (28)

**Figure 14:**
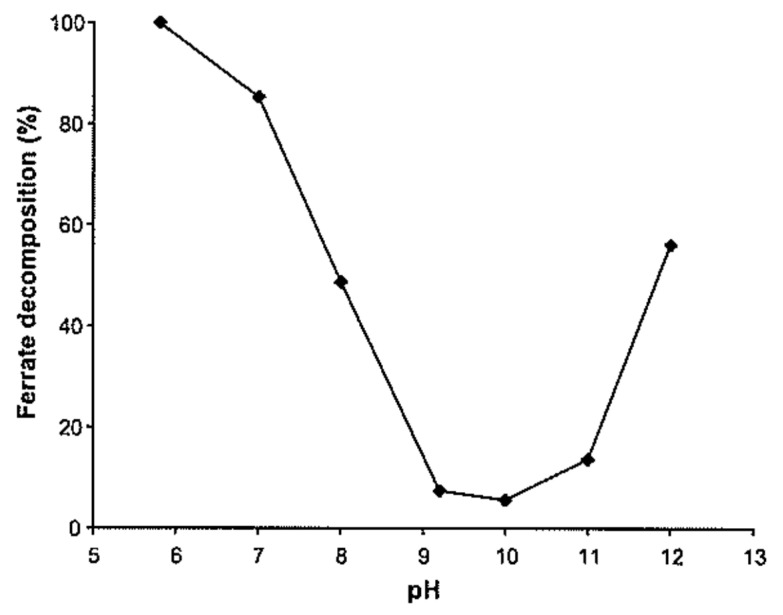
Effect of pH on the rate of decomposition of ferrate(VI) [118].

All the above assertions cannot be taken to mean that reactions exhibiting 2nd order kinetics will never take place between iron species in basic media. In fact, Rush & Bielski [140] asserted that, within the alkaline range 10 < pH < 12, a bimolecular reaction can occur only between **P_6,0_** and **P_6,1_**. However, at pH = 11, the mole fraction of **P_6,1_** in the Fe(VI) population is only 0.02%, and at pH = 12, it is ten times less than that (since pK_3_ = 7.2). If the rate equation is expressed as: Rate = *k* × [**P_6,0_**]*^a^* × [**P_6,1_**]*^b^* (a > 0, b > 0; if *a* = *b* = 1 the reaction is of true 2nd order), then the reaction will slow down considerably as the concentration of **P_6,1_** tends towards zero via deprotonation to become **P_6,0_**. This may partially explain the empirical observation of the slowing down of Fe(VI) decomposition as pH increases. Despite these findings, Graham et al. [118] reported an increase in the rate of ferrate(VI) decomposition from pH = 10 to 12, subsequent to a sharp decline from pH = 6 to 10. The initial concentration of the solutions was 0.25 mM, and its decomposition was monitored at 507 nm at 10 min from the introduction of the solution into the “UV-Vis” cell. Results are plotted, Figure 14. Carr et al. [141] set a good example in ferrate(VI) research when they took into account the parallel decomposition of Fe**^VI^**O_4_^2−^ during the oxidation of nitrite NO_2_^−^ ions. Control experiments were done separately to determine the rate of self-decomposition of Fe(VI) at pH = 14.9, at a solution temperature of 20 °C.

Oxidative hydrolysis is immediate on contact between ferrate(VI) and water; at pH = 6, decomposition of ferrate is complete after 10 min. Note that, in Figure 10, the rate of decomposition at pH = 12 (close to 60%) is greater than that at pH = 8 (which is 50%). This immediately suggests that freshly prepared ferrate(VI) solutions should be stored at pH = 10, the pH value at which decomposition is slowest (see Figure 10), although many synthetic chemists seem to prefer an alkaline pH rendered by 1 M NaOH, that of pH = 14. Even higher concentrations of alkali had been used, e.g., 10 M KOH by Licht et al. [24]. However, the number of OH^−^ ions present in the hydration sheath of the Fe(VI) anion will be limited by electrostatic repulsion. Do pH values drop so rapidly during redox reactions that so much caustic soda is required? It also needs be mentioned that there is scant rate data in the literature in the pH range 12 < pH < 14. Does decomposition stop abruptly at pH > 12, or does it proceed so sluggishly that the minute fraction of ferrate lost is of no consequence to the end-user between synthesis and application, no matter how long the oxidizing agent has been in storage?

Storage of solid K_2_FeO_4_ is best done in vacuum, and this is also an excellent opportunity to discover whether its crystalline and/or molecular structure and chemical composition will remain intact ad infinitum, so to speak. The headspace in the container can be monitored for gaseous O_2_ and temperature. Solid K_2_FeO_4_ (up to 99.5% pure) prepared by Licht et al. using the established wet chemical method of reacting NaOCl (soln.) with Fe**^III^**(NO_3_)_3_ lasted years [142]. On the other hand, Machala et al. discovered that K_2_FeO_4_ will decompose in warm and humid air [143].

Schmidbaur (2018) wrote an extensive review paper on the historical development of the discovery, analysis and chemistry of iron in its higher oxidation states, including the “non-existent” Fe(VIII) species FeO_4_. It is an extraordinary piece of scholarship. A panorama of many proposed pathways for the decomposition of ferrate(VI) in aqueous solution was provided. With illustrated elaborate reaction schemes, mechanisms for the decomposition of ferrate(VI) are portrayed and depicted in great detail [39].

### 6.3. A Self-Imposed Limitation of Fe(VI) Redox Reactions

It must be understood that waste treatment plants fall into two families; those single line facilities that treat waste at source and multiline facilities that can be at source (e.g., in-house waste treatment by an electroplating factory), but are more usually off-site commercial enterprises, taking liquid waste from a multitude of sources. The latter will take waste from diverse product lines and the contaminants will be equally diverse. They may range from relatively inert solids such as fine grits and fibres from various washing processes and include trace levels of fire retardants and pesticides; food preparation washings; acids containing dissolved metals; alkalis; phenols; peroxides; hypochlorite, organics and cyanides. (Often, a manufacturing firm mixes all its waste types in one tanker before delivery to waste processing plants, and is not a recommended practice). This list is not exhaustive and the wastes themselves will not be pure and may vary widely in concentration and frequency of arising. Whilst some of these wastes require side-line pre-treatment, ultimately almost all the wastes or their pre-treatment products will end up in the same main-line treatment process, the aim of which is to produce an inorganic solid cake of low toxicity for landfilling at an appropriate containment facility and a liquid of very low toxicity for further biological treatment. The latter may be at a treatment process on-site or off-site at a municipal sewage treatment works. In either case, discharged cake and final liquors must conform to disposal parameters and statutory limits, the significant requirements for licensing for waste disposal.

Therefore, the crux of the matter is not whether “Purple Iron” remains intensely purple at a particular pH subsequent to preparation. The pertinent issue of this Advanced Oxidation Process (AOP) is whether Fe(VI), Fe(V) and perhaps Fe(IV) species are capable, through redox reactions at any operational pH, transform the molecular structure of a hazardous substance to the extent that the end products pose substantially less or no immediate risk to environmental health, e.g., short of oxidizing an organic molecule exclusively to CO_2_ and H_2_O. In that respect, the time available for the detoxification mission to succeed is indeed limited. The essential redox steps for the task must be completed before any toxic descendent is incorporated into ferric flocs formed by the reduction Fe(VI) to Fe(III), with a substantial proportion of the flocs being formed as a result of rapid sacrificial decomposition of Fe(VI) at pH < 10 (i.e., in almost any type of water). Failing that, the sludges will be classified officially as hazardous substances themselves in most countries, and the problem of waste disposal remains unsolved. Instrumental methods of chemical analyses remain sine qua non to the quality control of solids to be disposed of by landfill.

Having alluded to the core issue of waste treatment by Fe(VI), namely, competitive redox vs. flocculation kinetics outlined in the paragraph above, it is appropriate, in the context of the overall discourse of the present work, to reflect upon the benefits of instrumental analysis of the structure and chemistry of Fe**^VI^**O_4_^2−^ and its relevance to environmental work. Recall the investigation of whether the ferrate(VI) ion is protonated in highly alkaline conditions (e.g., pH > 12) by Raman spectroscopy, and the answer was firmly in the negative [44]. The inference is that protonated ferrates in acidic media will give spectra from distorted tetrahedrons. By combining spectral data with speciation diagrams (e.g., Figure 8 which is in essence the end result of Henderson-Hasslebach calculations, e.g., Equation (14)), together with determinations of the rates of decomposition of ferrate(VI) by “UV-Vis” at different pH values, detection of ^18^O in evolved oxygen [45] and other established methods such as cyclic voltammetry to follow redox reactions, a phenomenological model of the decomposition of ferrate(VI) can be obtained upon which redox reactions involving water pollutants can be built. The best pH value for treatment can then be estimated and tested. Research in waste treatment by Fe(VI) is an exercise in process optimization.

### 6.4. The Solubility Products of Fe(II) and Fe(III) Hydroxides

Discussion of water treatment by Fe(VI) will not be complete without addressing the Fe(II) and (III) states. Attempts to determine the solubility and solubility product *K_sp_* of iron(II) hydroxide in the laboratory since the 19th century have been beset with difficulties, and as a result, uncertainties in experimental results abound. The situation had still not been resolved satisfactorily by the mid 20th century, at which time the numerical value of *K_sp_* had undergone revision by 8 orders of magnitude, from 10^−21^ to 10^−13^ [144]. One of the difficulties of determining the solubility and solubility product of ferrous hydroxide is the ease with which Fe(II) can be oxidized to Fe(III). Determination must be carried out in de-aerated water and inert atmosphere. The 84th edition (2003) of “CRC Handbook of Physics and Chemistry” lists a value of 4.87 × 10^−17^ mol^3^ dm^−6^ [145] and is consistent through its many editions; for the pure sake of illustration, the value given in the CRC Handbook is used here for the calculation of the pH value at which 1 μM of ferrous ions will form Fe(OH)_2_. Therefore,

*K_sp_* = [Fe^2+^] × [OH^−^]^2^ (29)

Therefore, *K_sp_* = (1 × 10^−6^) × [OH^−^]^2^ = 4.87 × 10^−17^.

Rearranging and solving the quadratic equation gives:

[OH^−^] = 2.2 × 10^−5.5^ mol dm^−3^

pOH = −log_10_ [OH^−^] = −log_10_ (2.2 × 10^−5.5^) = 5.1

The solubility product for water *K_w_* is such that:

Log_10_*K _w_* = pH + pOH = 14 (30)

By definition, pH = 14 − pOH = 14 − 5.1 = 8.9. Therefore, with [Fe^2+^] = 54 μM, the choice of concentration of McBeath et al. [3] in the electrochemical synthesis of Fe**^VI^**O_4_^2−^, ferrous hydroxide Fe(OH)_2_ is expected to form at pH = 7.8 by the same calculation above, but if the solution mixture is phosphate buffered at pH = 7.1, insoluble iron(II) hydroxide is unlikely to form. In this instance, PO_4_^3−^ and HPO_4_^2−^ ions did not seem to have precipitated the ferrous ions themselves. Note that ferrous phosphate is insoluble, log_10_*K_sp_* of the mineral vivianite is of the order −36. Moreover, it pays to be aware that species such as “ferrous biphosphate” which has the formula (Fe**^II^**)PO_3_(OH), and ferrous hydroxy phosphate [(Fe^2+^)_2_(OH^−^)(PO_4_^3−^)]^0^ are only sparingly soluble in water. Electrochemical methods to synthesize ferrate(VI) were reported by Mellor as early as 1934 [146], and have regained popularity recently.

It is a fair question to ask whether direct electrolysis of a potential water pollutant (e.g., electrolysis of Cu-EDTA which arises from the manufacture of Printed Circuit Boards [147]) will save the effort of synthesizing an oxidant by an electrochemical method before treatment begins. A fair answer is that, akin to all other wastewater treatment methods, electro-oxidation produces unintended results once in a while (but this does not negate the validity of the treatment of liquid wastes by electrolysis as a general method). Applicability is specific to the compound of interest. For example, Gattrell & Kirk [148] attempted to electrolyze phenol but obtained free radicals of the compound instead. The radicals then polymerized into a substance no less toxic than phenol itself, a rare and interesting case. (See [9] for the treatment of pentachloro-phenol by ferrate(VI)).

In the quantitative analysis of Fe^2+^ (aq) ions, elimination of dissolved O_2_ and any contact with air during material transfer between containing vessels, e.g., during the making up of standard solutions, are essential. Any oxidizing agent in the reaction mixture will facilitate the formation of Fe^3+^ ions which may precipitate in due course. Moreover, atmospheric CO_2_ dissolves in water to furnish CO_3_^2−^ and HCO_3_^−^ ions, giving rise to iron carbonates which are insoluble at pH = 9. The presence of these unwanted contaminants will no doubt affect the position, shape and intensity of peaks during the identification of ferrate(VI).

In the case of ferric ions, a calculation with 1 μM Fe^3+^ ions, using *K_sp_* = [Fe^3+^] × [OH^−^]^3^ = 2.79 × 10^−39^ [145] shows that entities of iron(III) hydroxide will form at pH = 3.1, in the acidic region. From the example above, assuming that the Fe(II) → Fe(III) transition, buffered at pH = 7.1, is stoichiometrically complete, then for [Fe^3+^] = 54 μM,

[Fe^3+^] × [OH^−^]^3^ = (54 × 10^−6^) × (10^−6.9^)^3^ = 5.4 × 10^−25.7^ > 2.79 × 10^−39^ (*K_sp_*).

Therefore, Fe(III) is likely to be in colloidal form while being oxidized to higher states. However, the presence of an oxidizing environment will probably not halt these three rapid hydrolysis steps:

Fe^3+^ + H^+^(OH)^−^ ⇌ [Fe**^III^**(OH)]^2+^ + H^+^ (31)

[Fe**^III^**(OH)]^2+^ + H^+^(OH)^−^ ⇌ [Fe**^III^**(OH)_2_]^+^ + H^+^ (32)

[Fe**^III^**(OH)_2_]^+^ + H^+^(OH)^−^ ⇌ Fe(OH)_3_ + H^+^ (33)

These calculations for ferrous and ferric ions show that, the higher the concentration of the metal ions present, the lower the pH value is required to keep them in solution. The entities Fe(OH)_2_ (aq) and Fe(OH)_3_ (aq) are seldom mentioned in literature. However, during an investigation of the hydrolysis of FeCl_3_ in dilute solution, Lamb & Jacques [149] discovered that any “ferric hydroxide” present would be in super-saturation, but it is not known whether discrete molecules of Fe(OH)_3_ were present in true solution, which “dimerize” on aging to give the hydrated iron(III)oxide Fe_2_O_3_·3H_2_O, or still larger aggregates of sub-colloidal size (<1 nm), or whether the super-saturated system is inherently unstable and tends towards phase change. Furthermore, it was discovered that the higher the concentration of total iron, the faster the onset of precipitation (when the solubility product is exceeded).

In the speciation of Fe(II) and Fe(III) as a function of pH, a computer model should at least include the self-ionization of the hydroxides [150], so that:

Fe**^II^**(OH)_2_ (aq) ⇌ Fe**^II^**(OH)^+^ + OH^−^ (*K_eqm_* = 2 × 10^−5^) (34)

Fe(OH)_3_ (aq) ⇌ Fe**^III^**(OH)_2_^+^ + OH^–^ (*K_eqm_* = 2.5 × 10^–8^) (35)

When precipitation is complete, equilibria are set up between the solid and aqueous phases, so that:

Fe(OH)_2_ (c) ⇌ Fe^2+^ + 2 OH^−^ (*K_eqm_* = 1.8 × 10^−15^) (36)

Fe(OH)_3_ (c) ⇌ Fe^3+^ + 3 OH^−^ (*K_eqm_* = 1.0 × 10^−38^) (37)

(Note that the numerical value *K_eqm_* = 1.0 × 10^−38^ in Eqt. 37 is just one of many reported values.)

Lamb & Jacques [149] estimated that the maximum solubility of Fe(III) hydroxide in water will not exceed 2 nM in clarified water subsequent to precipitation. This is 1 ppb of Fe(OH)_3_ in suspension, or 0.5 ppb with respect to iron atoms [149]. On acidification, this concentration or iron in aqueous solution is still detectable by ICP-OES (Inductively Coupled Plasma—Optical Emission Spectroscopy), as Perkin-Elmer reported that the Method Detection Limit for iron is 0.3 ppb [151]. The problem is, in a concentrated solution, suspended particles in aqueous mixtures can cause severe Rayleigh scattering. When particle sizes are smaller than the wavelength of light, such as colloids, ferric flocs and aggregates in the process of sedimentation or creaming, incident photons will be scattered elastically. Rayleigh scattering will affect the measured absorption in a “UV-Vis” spectrum since the scattered photons will not reach the detector of the spectrometer and therefore will be interpreted erroneously as absorbed light by the instrument. (This does not apply to ICP-OES since the samples to be analysed are well filtered to obtain homogeneous solutions, the only phase of interest in this particular spectroscopy). This severely limits the time available for precise analysis via the visible spectrum. Wood’s comment [68] on the absorption coefficients of obtained by Kaufman & Schreyer [69] was already mentioned.

Some general remarks about Pourbaix diagrams need be made. When a Pourbaix diagram is used to establish the predominance of a species, it must not be inferred that a particular species cannot possibly exist outside its stability region, e.g., in the complex redox reactions of Fe(VI) introduced so far, it is possible for species such as Fe**^VI^**O_4_^2−^ (aq) and Fe(OH)_3_ (aq) to be present in homogeneous solution together, albeit the coexistence being transient. The argument is the same for the transitory nature of Fe(V) and Fe(IV) species, and all the protonated forms of Fe(VI), Fe(V) and Fe(IV). Thermodynamically, the boundaries of a stability field are best interpreted as contours at which a unique species and an alternative may well be equally important. Indeed, the octahedral species [Fe**^III^**(H_2_O)_6_]^3+^ can exist in multiple equilibria with its hydroxy substitutes such as [Fe**^III^**(H_2_O)_5_(OH)]^2+^ and [Fe**^III^**(H_2_O)_4_(OH)_2_]^+^, but these species are seldom displayed on a Pourbaix diagram for iron. Non-stoichiometric Fe(II, III) phosphates are stable salts, but the speciation rarely appears on a Pourbaix diagram. Note as well that the boundary between a solid phase and a solution phase will depend on the concentration chosen for the aqueous species. It is reasonable to assume that beyond the confines of its stability region, a species no longer dominates the population but becomes less prevalent the further conditions are adrift from those which define stability.

## 7. Further Work

### 7.1. Acquire the Inelastic Neutron Scattering (INS) Spectra of Ferrate(VI)

This is the third type of vibrational spectroscopy available to analysts and provides a complementary method to Raman and infrared spectroscopies for the scrutiny of molecular vibrations. One great advantage of INS is that there are no selection rules which forbid certain bands to appear, and all vibrations are observed. INS is also very sensitive to isotopic substitution, and will be an ideal tool to follow reaction pathways were it possible to synthesize the ferrate(VI) ion with exclusively ^18^O atoms, i.e., the deep purple [Fe**^VI^**(^18^O)_4_]^2−^ ion in NaOH solution. The task at hand is to test the hypothesis forwarded by Griffith ([44,78]), as to whether the ferrate(VI) in K_2_FeO_4_ is of *C_s_* “site-symmetry”, downgraded from *T_d_*. There is precedence of utilizing INS in the solving of mysteries regarding point groups. Stirling et al. [152] combined all three vibrational spectroscopies and eliminated the possibility of a *D_∞__h_* designation for the two caesium salts CsHCl_2_ and CsDCl_2_ and instead allocated two new point groups, namely *C*_2*v*_ or *C_s_*. The investigation is worth attempting and will open up a new understanding of vibrational modes for all tetraoxo ions with metals in their highest oxidation states, including Fe(VII) [103] which was introduced in the present review.

The microcrystalline structures of potassium sulphate, chromate and ferrate were established to be “isomorphous” in the late 19th century, in 1892 [153]. It is time to obtain their INS spectra and compare the symmetries of the anions.

Anyone interested in utilizing Density Function Theory (DFT) in the determination of normal modes of vibration, and of band intensities in infrared and Raman spectra should first consult a paper by Horvath & Gordon [154]. Methods to extract data from the Franck-Condon region are illustrated and discussed. There are many successful cases of assignment and interpretation of infrared and Raman spectra with the aid of DFT. For example, Molchadova et al. (2021) simulated the infrared spectra of the complex Co_3_(BO_3_)_2_ by ab initio calculations of the lattice dynamics, which led to normal-mode assignments [155]. These M_3_(BO_3_)_2_ complexes bear magnetic properties with many useful applications. On another occasion, Zajac et al. (2019) copper phytate complexes were studied by a combination of ATR (Attenuated Total Reflection)/Infrared, FT-Raman, UV–Vis, EPR spectroscopies, magnetic measurements and DFT calculations [156]. Studying phytic acid is important because the compound has adverse medical implications. Phytic acid is a macromolecule with the formula C_6_H_18_O_24_P_6_ and is a six-fold dihydrogenphosphate ester of inositol, also called inositol polyphosphate. In plants, phytic acid acts as the main store house of phosphorus. All edible seeds, grains, legumes and nuts contain it in varying quantities, and small quantities are present in roots and tubers. At human physiological pH, the phosphate groups are partially ionized. The degree of ionization is a function of the pK_a_ of the groups, and phytate are organic ligands which can form chelated complexes with metallic ions. This complex-formation attenuates the absorption of calcium, iron and zinc ions and may therefore promote mineral deficiencies for the organism. There is little surprise that phytic acid is infamously known as an anti-nutrient. Another case study worth reading is that of [Ru(bpy)_3_]^2+^ used in sensors and solar cells. A high-resolution mathematical scheme which included Duschinsky coupling, solvent effects and anharmonicity modelled vibrational resonance Raman spectra [157]. One type of question to which DFT calculations can help provide an answer can be illustrated by the investigation of the difference between the structurally similar chelated complexes Ni(II)-L and Zn(II)-L, where the ligand L = O,O-diethyldithiophosphate. The largest difference could be one in symmetry, in the vibrational modes of central part of the formula unit PS_2_(M**^II^**)S_2_P. Within this unit, the Ni(II) complex exhibits D_2h_ symmetry while for Zn(II), it is D_2d_ [158]. One of the most satisfactory collaborations between laboratory experimentation and computational methods is the case of Al_4_SiC_4_ [159]. The vibrational spectra were analysed by DFT calculations, resulting in the assignment of all Raman modes and most infrared modes.

The next challenge for Fe(VI) research is the acquisition of the Inelastic Neutron Scattering (INS) spectra of ferrate(VI) and systematic analysis of results by computational methods, leading to assignment of all bands and origins of their electronic transitions.

### 7.2. Re-Investigate the Rate of Ferrate(VI) Self-Decay from Mild to High Alkalinity

Using UV-Vis spectrophotometry and the ABTS reaction (and/or other appropriate techniques), determine the decomposition rate under the following conditions. Check if the rate of decomposition of ferrate(VI) increases with alkalinity.

(a) pH = 9, 10, 11, 12, 13 and 14.
(b) Concentrations of ferrate = 1, 5, 10 and 20, 50 mM of analytical grade K_2_FeO_4_.
(c) Temperatures of aqueous solutions = 5 °C, 15 °C, 25 °C, 35 °C.

### 7.3. Investigation of the Effect of the Metal Ions on Ferrate(VI) Decomposition

The treatment of water by ferrate(VI) for potable purposes warrant investigation since natural bodies of water can be “hard” or “soft”. The work of Chen et al. [122] had been introduced earlier in this review; the presence of Ca^2+^ accelerates the decomposition of FeO_4_^2−^ in alkaline solution. Whether the resulting calcium diferrate(IV) complex ion performs better than FeO_4_^2−^ as a treatment agent itself requires laboratory studies. The same research should apply to industries which draw upon natural waters for various uses. Moreover, the wastewaters can contain Ca^2+^ ions due to commercial chemical processes themselves. On a related subject, sodium hypochlorite NaOCl affects the oxidations Ni(II)→Ni(III) and Co(II)→Co(III) with ease in alkaline solutions, precipitating the metal ions as insoluble oxyhydroxides M**^III^**O.OH (these are not peroxides). Will ferrate(VI) perform the same task effectively in alkaline? Tests in the laboratory can find out.

### 7.4. Investigation of Components of Iron Oxide-Hydroxide Sludges as Part of the Research

Relatively ignored are the characteristics of the particles and precipitates formed as a result of the Fe(VI) → Fe(III) reduction. Goodwill et al. [160] demonstrated that these are the hydrated iron(III) oxide Fe_2_O_3_, and not Fe(OH)_3_ as one may instinctively assume. This will make a difference to the adsorptive and coagulative ability of the “iron floc” in the treatment of wastewaters, in terms of availability of total surface area, type and density of electric charges on the surface of these particles, and the mechanical robustness of the floc. Moreover, scant attention has been paid to the fact that hydrated Fe_2_O_3_ can play the role of an oxidizing agent [161] and this is worth investigating further. In 2020, there has been a lot of attention devoted to the “activation” of Fe(VI) by reductants such as SO_3_^2−^ before the actual treatment process itself, the implications for downstream processing of wastewaters have been examined and discussed by Bzdyra et al. [162]. (The half-equation requires the sacrifice of 2 moles of electrons per mole of sulphite (SO_3_^2−^) for the activation, but the benefit is that the more reactive Fe(IV and/or V) states may be attained quicker in aqueous solution.) Investigation into the physical-chemical nature of the iron nanoparticles, with or without “activation”, should continue as these have significant technological and economic implications.

There has been little reported about the chemical composition of the de-watered filter cake obtained after treatment with Fe(VI). The most important characteristics to be considered is whether toxic materials and their breakdown components are still adsorbed and enmeshed in the large three-dimensional network of ferric flocs subsequent to dewatering. In an effort to identify toxic products which resulted from treating PPCP (pharmaceuticals and personal care products) wastes, Barisci & Dimoglo [163] described the mechanistic pathways of the reactions between ferrate(VI) and antibiotics, analgesics, β-blockers, lipid regulators, anti-psychotics and cytostatic drugs in detail, a rarity in the literature of wastewater science. This area of knowledge should be added to the “gaps” highlighted by Professor Guan Xiahong (Tonji University, Shanghai, China) and colleagues from various institutes (2021) in their review of ferrate(VI) [164].

In general, the type and quantity of hazardous materials (i.e., its total toxicity) present in a dried cake or sludge will decide whether its disposal is allowed by local authorities. Therefore, in the laboratory, it may be necessary to leach a sample of the filter cake by non-oxidizing acids such as HF (used to extract components from geological samples) and analyse the leachate by a combination of instrumental methods as appropriate. The U.S. Environmental Protection Agency (EPA) has produced voluminous instruction manuals on how to perform these tests, depending on the specific requirements. Production of a disposal solid material should be a criterion of successful treatment by Fe(VI) on an industrial scale. On that note, instrumental methods of identification and quantification of hazardous substances in liquid and solid phases remain, more than ever, indispensable.

### 7.5. Storage of Solid K_2_FeO_4_

This is best done in vacuum, and this is also an excellent opportunity to discover whether its crystalline and/or molecular structure will remain intact ad infinitum, so to speak. The headspace in the container can be monitored for gaseous O_2_ and temperature. Solid K_2_FeO_4_ (up to 99.5% pure) prepared by Licht et al. using the established wet chemical method of reacting NaOCl (soln.) with Fe**^III^**(NO_3_)_3_ lasted years [142]. On the other hand, Machala et al. discovered that K_2_FeO_4_ will decompose in warm and humid air [143], a situation to be avoided.

### 7.6. Further Reading: Latest Theoretical Developments on Chemical Bonding

A modern theory in chemistry to explain observed molecular structure and bonding behaviour has been propounded. It is called Inverted Ligand Field Theory. Proponents offer an alternative explanation for the spectroscopic properties and chemical reactivities of “high” valent and “late” 3*d* transition metal complexes. For the theory to work, it demands that the energies of ligand orbitals to be higher than those of the *d* orbitals of the metals. The foundational moiety for the theory was [Cu(CF_3_)]^−^ (the copper nitrene complex ion) with a formal Cu(III) centre, but inverted theorists demonstrated that it is more suitably being described as Cu(I). The M.O. description is one in which the anti-bonding MOs are based on the AOs of the ligands. X-ray absorption spectroscopy (XAS) revealed no *d*-*d* transitions in the near-infrared and visible regions. Simulation of the X-ray spectrum by time-dependent Density Function Theory lent support to the idea of an inverted ligand field, with copper’s *d* orbitals fully occupied while a hole exists in a ligand MO. Hoffman et al. (2016) projected that the development of the inverted theory will focus on copper and zinc [165], while Betley et al. synthesized a “copper-supported triplet nitrene complex” [166], i.e., research is still focused on copper as of 2019, but there has been investigations into other transition elements, e.g., the oxidation of Ni(II) to Ni(IV) with aryl electrophiles enabling “Ni-mediated aryl-CF_3_ coupling” [167]. Traditionally, zinc is not considered a transition element, but that is hardly the issue here. As far as chemical bonds are concerned, it may be possible to equate copper’s d^9^ configuration to that of zinc’s full quota of electronic arrangement. The potential for research is huge. There is a myriad of oxygenated compounds with metals in their higher oxidation states which have not been analysed by computational methods. A case in point is praseodymium. Dingle (2018) included three species in his review of the element’s chemistry [168], namely, [Pr**^V^**O_2_]^+^, NPr**^V^**O and [NPr**^IV^**O]^−^. Will the assignment of these oxidation states change? The saga continues. Scientific revolutions described by Thomas Kuhn [169] may just occur. Still, the stoichiometry of electron-transfer reactions in well-known redox pairs such as MnO_4_^−^/I^−^ and OCl^−^/Ni^2+^, and also in the disproportionation of Am (VI) in alkaline solutions into Am (V) and the extremely unstable Am (VII), have to be accounted for by any new theory.

In July 2021, personal communications between the Nobel Laureate Professor Roald Hoffmann and his colleague Professor Kyle M. Lancaster (Cornell University, Ithaca, NY, USA) and the authors of the present review paper came to fruition. By 2 July 2021, Hoffmann’s group has yet to examine the [FeO_4_]^2−^ moiety by the tools of computational chemistry. Nevertheless, our dialogue (which commenced on that day) about Fe(VI) seemed to have enthused Hoffmann and Lancaster on the subject. Lancaster performed a Density Function Theory (DFT) calculation on [FeO_4_]^2−^ on our behalf, for which we are extremely grateful. The results of this calculation was related to us on 8 July 2021 by Hoffmann (per verbatim), “We expected significant Fe(3d)-O(2p) mixing, but probably no inversion. Kyle (Lancaster) reports: ‘The *t*_2_ set of orbitals in [FeO_4_]^2−^ have about 45% Fe 3d in them with some minor (ca 5% Fe 4p) admixture. The occupied *e* set is about 55% Fe 3d’. Thus mixing, but I’m hesitant to label it a case of clear inversion”. While the electronic structure is not “inverted” in the strict sense, it does not necessarily imply that the Fe(VI) assignment should stand. On 22 August 2021, Lancaster commented further (per verbatim), “With 6 holes at 45% Fe 3d and 2 holes at 55% Fe, the calculated d-count for Fe is effectively d^6^, which implies an Fe(II) with substantial hole character delocalized over the four O-donors”. These are fascinating developments, and warrants further exploration of how they can be related to the UV-Vis, IR, Raman and Inelastic Neutron Scattering spectra, and also to the chemical versatility of “Purple Iron” as an oxidant.

The two Cornell chemists also recommended the following literature for further reading: Barandiaran et al. (already cited as reference [65] and discussed at length in this work); Wolfsberg & Helmholtz, on the spectra and electronic structure of tetrahedral ions [170]; Ballhausen & Gray, on Molecular Orbital Theory [171], which the authors of the present review had deployed to explain the visible spectrum of ferrate(VI); Schmidbaur & Schwarz (2021), on Group VIIB metal-trioxo-halides, MO_3_X [172], a sequel to another paper they published in 2021 on MnO_3_F [114].

## 8. Conclusions

Critique of literature pertains to the realm of epistemology. In this work, the origin, nature, validity and limitation of some spectroscopic methods, spectral knowledge and thermodynamic narratives with regards to the tetraoxoferrate(VI) ion are scrutinized. Specifically, the origins of the bands in the Ultraviolet-Visible (“UV-Vis”), Infrared and Raman spectra of Fe**^VI^**O_4_^2−^ were described. There is consensus in the literature that the intense purple colouration of the ion is a result of ligand-to-metal charge transfer processes. The Fe**^VI^**O_4_^2−^ ion in highly alkaline solution retains its tetrahedral shape and also its *T_d_* (high symmetry) point group. Discussions in the literature concerning downgrading to the *C_s_* point group to be the “site-symmetry” of ferrate(VI) in crystals were reviewed; this remains a hypothesis to be tested.

The discrete Fe**^VI^**O_4_^2−^ ion is thermodynamically stable in a small region on the Pourbaix diagram at pH > 10 and below the water oxidation line. Literature has documented situations in which the ion can decompose in the region 10 < pH < 12, although no kinetic data is available for decomposition at pH > 12. The preparation of ferrate(VI) in solutions of pH = 14 seems justified by virtue of the position of Fe(VI) in the Pourbaix diagram for iron. Two mechanisms for the decomposition of ferrate(VI) are recognized in the literature. In acidic conditions, di-iron complexes are first formed which then oxidize solvent water, while in alkaline conditions self-decay of the Fe**^VI^**O_4_^2−^ moiety is apparent. Between the extremities of acidity and alkalinity both mechanisms are at play; the dualism is on clear display in solutions at near-neutral pH conditions. Insolubility of Fe(II) and Fe(III) hydroxides causes phase separation in aqueous systems. It is documented in literature that Rayleigh scattering of incoming “UV-Vis” photons by suspended solids usually give higher readings of absorbance than expected. A gap of knowledge has also been identified, namely, there is almost no work reported on investigation of toxicities of materials encapsulated in ferric flocs subsequent to treatment by Fe(VI). Potentially, this can be a serious issue if such toxic materials are released into the natural environment.

The relevance of integrated theoretical and instrumental (experimental) analysis to the aqueous chemistry of Fe**^VI^**O_4_^2−^ and therefore to its environmental applications has been borne out in this review. Mentioned in this work is the use of Raman spectroscopy to investigate whether the ferrate(VI) ion is protonated in highly alkaline conditions (e.g., pH > 12), and it was found not [63]; the implications are that protonated ferrates at lower pH values will give different spectra of distorted tetrahedrons. Therefore, by combining vibrational spectroscopic data with speciation models, rates of decomposition of ferrate(VI) by “UV-Vis”, provenance of ^18^O in evolved gaseous oxygen O_2_ (from the water solvent or the ferrate ion itself) [68] and other kinetic data, a mechanistic picture of the “self-decomposition” of ferrate(VI) has begun to emerge. Redox reactions involving water contaminants can then be superimposed on this foundational scenario from which optimal operational conditions and parameters can be derived.

## Figures and Tables

**Table 1:** Raman and infrared peaks of solid K_2_FeO_4_ and Na_2_FeO_4_ (cm^−1^).

Authors	Tarte/Nizet [84]	Griffith [44]	Gonzalez/Griffith [78]	Maghraoui et al. [86]
Year	1963	1966	1972	2015
Solid compound	K_2_FeO_4_	K_2_FeO_4_	K_2_FeO_4_	Na_2_FeO_4_
*ν*_1_ (Raman)	-	-	830(10)	-
*ν*_1_ (i.r.)	782	779 *m* (but disappears in D_2_O)	-	~750
*ν*_2_ (Raman)	-	-	336(½)	-
*ν*_2_ (i.r.)	Possible association with *ν*_4_ (340, 322)	-	340 *m*	(not reported)
*ν*_3_ (Raman)	-	-	796(6) 786(1)	-
*ν*_3_ (i.r.)	809 (singlet) 825 (small shoulder)	827 *sh* 810 *s* 796 *s* (merges in D_2_O as one band at 800) *s*	816 *w* 796 *vs* 780 *m*	825 *s* (The 870 peak in [61] is actually from K_2_CrO_4_).
*ν*_1_ + *ν*_3_ (Raman)	-	-	-	-
*ν*_1_ + *ν*_3_ (i.r.)	(not reported)	1570 *w*	(not reported)	(not reported)
*ν*_4_ (Raman)	-	-	318(2) 312(3) 307(1)	-
*ν*_4_ (i.r.)	340 *w* 322 *w*	339 *m* 320 *s* (only the 320 peak remains in D_2_O)	324 *w* 319 *vs* 311 *w*	(not reported)
*ν*_2_ + *ν*_4_ (Raman)	-	-	(not reported)	-
*ν*_2_ + *ν*_4_ (i.r.)	(see *ν*_4_ above)	-	(not reported)	(not reported)
Other strong bands (Raman)	-	(see two boxes to the right)	840(2)	-
Other strong bands (i.r.)	(not reported)	(see two boxes to the right)	620 *s* 297 *w*	1140, 950, 930, 860, 620 (all suphate related).

Note: *m* = medium strong peak; *sh* = sharp peak; *s* = strong peak; *w* = weak peak. The wavelength reported as “830(10)” means that, within a few scans lasting 1 to 10 min, the most intense band (judged mostly by the eye) is the one collected at the 10th minute. The same notation applies to other wavenumbers in Table 1 and Table 2.

**Table 2:** Raman peaks of K_2_FeO_4_ in aqueous solution (pH~13.6) ([44,78]).

Vibration Mode	Wavenumbers (cm^−1^)	Polarization
*ν*_1_	832(10)	Polarized
*ν*_2_	340(3)	Depolarized
*ν*_3_	790(6)	Depolarized
*ν*_4_	322(5)	Depolarized
